# Nir1-LNS2 is a novel phosphatidic acid biosensor that reveals mechanisms of lipid production

**DOI:** 10.1101/2024.02.28.582557

**Published:** 2024-02-28

**Authors:** Claire C. Weckerly, Taylor A. Rahn, Max Ehrlich, Rachel C. Wills, Joshua G. Pemberton, Michael V. Airola, Gerald R. V. Hammond

**Affiliations:** 1. Department of Cell Biology, University of Pittsburgh School of Medicine, Pittsburgh, PA, USA; 2. Department of Biochemistry and Cell Biology, Stony Brook University, Stony Brook, NY, USA; 3. Section on Molecular Signal Transduction, Program for Developmental Neuroscience, Eunice Kennedy Shriver NICHD, National Institutes of Health, Bethesda, MD, USA

## Abstract

Despite various roles of phosphatidic acid (PA) in cellular functions such as lipid homeostasis and vesicular trafficking, there is a lack of high-affinity tools to study PA in live cells. After analysis of the predicted structure of the LNS2 domain in the lipid transfer protein Nir1, we suspected that this domain could serve as a novel PA biosensor. We created a fluorescently tagged Nir1-LNS2 construct and then performed liposome binding assays as well as pharmacological and genetic manipulations of HEK293A cells to determine how specific lipids affect the interaction of Nir1-LNS2 with membranes. We found that Nir1-LNS2 bound to both PA and PIP_2_ *in vitro*. Interestingly, only PA was necessary and sufficient to localize Nir1-LNS2 to membranes in cells. Nir1-LNS2 also showed a heightened responsiveness to PA when compared to biosensors using the Spo20 PA binding domain (PABD). Nir1-LNS2’s high sensitivity revealed a modest but discernible contribution of PLD to PA production downstream of muscarinic receptors, which has not been visualized with previous Spo20-based probes. In summary, Nir1-LNS2 emerges as a versatile and sensitive biosensor, offering researchers a new powerful tool for real-time investigation of PA dynamics in live cells.

## Introduction

Phosphatidic acid (PA) is a truly versatile lipid, with parallel activities as a vital metabolic intermediate, a second messenger, and a determinant of unique membrane properties (Zhou et al., 2023). PA serves as a precursor for lipid species such as diacylglycerol (DAG), lysophosphatidic acid (LPA), and CDP-diacylglycerol (CDP-DAG), each of which is used in its own signaling and metabolic pathways (Thakur et al., 2019). PA regulates the localization and function of various enzymes such as phosphatidylinositol 4-phosphate 5-kinase (PIP5K) (Cockcroft, 2009), mTOR (Frias et al., 2023), ERK (Zhang et al., 2014), and Hippo (Han et al., 2018). Finally, PA controls membrane architecture by inducing negative membrane curvature (Zhukovsky et al., 2019), thereby playing a role in membrane trafficking (Zeniou-Meyer et al., 2007; Tanguy et al., 2021). Due to these multiple roles, PA and its associated regulatory and effector enzymes have been identified as therapeutic targets in a variety of diseases such as cancers, neurodegenerative diseases, and hypertension (Brown et al., 2017; Bruntz et al., 2014; Castagna et al., 1982; Cooke and Kazanietz, 2022; Dhalla et al., 1997; Fazio et al., 2020; Kato et al., 1992; Sakane et al., 2008; Tappia and Singal, 2009; Thakur et al., 2019). Despite the role of PA in a multitude of cell functions and diseases, the regulation of PA is not fully understood. This is in part due to a lack of high-affinity tools available to study PA in live cells.

PA is produced at the plasma membrane (PM) through two interrelated pathways: activation of phospholipase C (PLC) and diacylglycerol kinases (DGKs) (Kadamur and Ross, 2013; Shulga et al., 2011) and activation of phospholipase D (PLD) by protein kinase C (PKC) (Selvy et al., 2011). These pathways are thought to be stimulated consecutively as PLC activity increases DAG and intracellular Ca^2+^ levels, which then in turn activates PKC and PLD. While recent click-chemistry fluorescent lipid reporters have shown PLD activation by PLC signaling, the role of PLD in producing endogenous PA downstream of the PLC pathway is still unclear (Liang et al., 2019). This highlights the need for better tools to study PA production in real time within living cells.

The most robust way to study lipids in live cells is through genetically encoded lipid biosensors (Maekawa and Fairn, 2014; Wills et al., 2018; Hammond et al., 2022). These sensors are a fluorescently tagged effector protein or domain that binds specifically to a lipid of interest and labels it in intracellular membranes. However, biosensors need to be carefully characterized to avoid misinterpretation of lipid dynamics. Our lab has previously defined three criteria that we consider to be crucial for a lipid biosensor to meet: is the biosensor specific for the lipid of interest? Is the membrane localization of the biosensor dependent on that lipid? And, is the lipid of interest sufficient to localize the biosensor to membranes? (Wills et al., 2018).

A variety of effector PA binding domains (PABDs) have been characterized with the goal of utilizing them as PA biosensors, including *S. cerevisiae* Opi1 (Hofbauer et al., 2018), PABD-PDE4A1 (Baillie et al., 2002), PABD-Raf1 (Ghosh et al., 2003), and the N-terminus of alpha-synuclein (Yamada et al., 2020). However, these all display somewhat limited PA-membrane binding, respond to additional stimuli such as membrane curvature or Ca^2+^ flux, or haven’t been characterized in cells with endogenous PA levels (Kassas et al., 2017, 2012).

**Figure 1.**
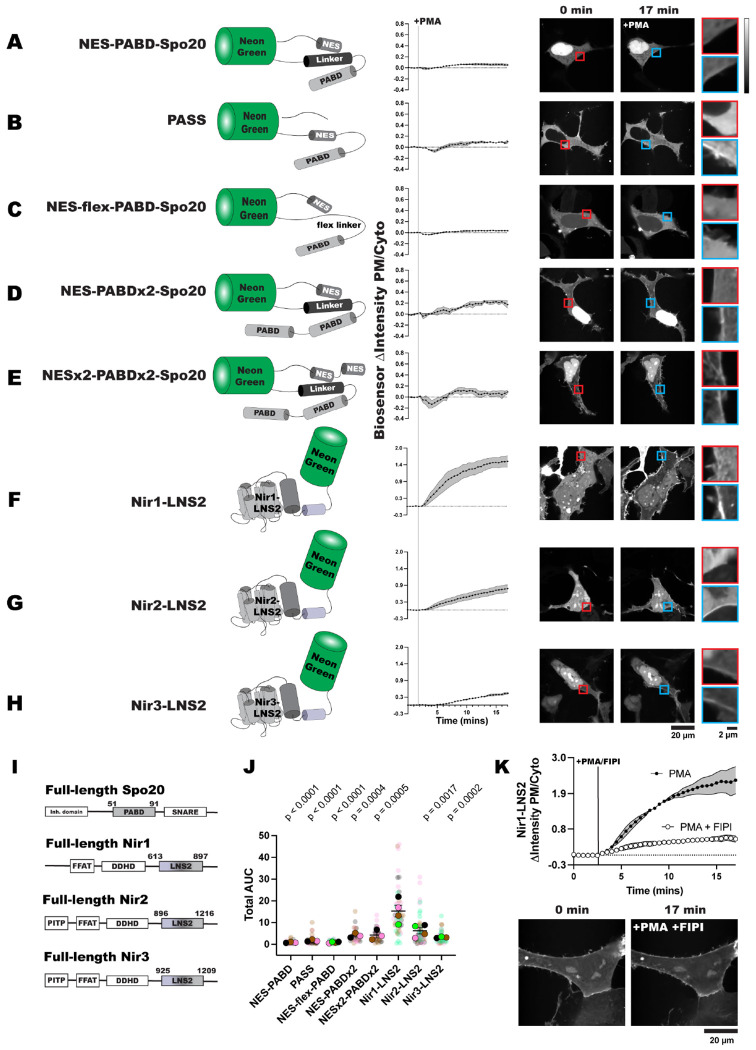
Nir1-LNS2 is highly sensitive to PA. **(A-E)** Phosphatidic acid biosensors were made from the phosphatidic acid binding domain (PABD) of Spo20 with added nuclear export sequences (NES) and various linker sequences. Alpha helices are shown by thin cylinders while unstructured regions are shown as lines. **(F-H)** Novel biosensors were designed using the LNS2 domain of the Nir family of proteins. Sensors translocate to the PM after PLD activation with 100 nM PMA in transfected HEK293A cells. The red inset shows the PM intensity of the sensor before PMA stimulation, and the blue inset shows the PM intensity after PMA stimulation. Data shown is the grand mean of 3-4 experiments ± SEM. **(I)** Schematics of full-length Spo20 and Nir proteins. **(J)** Total area under the curve (AUC) for the biosensor responses in A-H was calculated using Prism and compared to the Nir1-LNS2 AUC. The small circles indicate the AUC of individual cells (n=26-52). The large circles show the average AUC for each experimental replicate (n=3-4). Cells in each replicate are color coded accordingly. Statistics were calculated with a post-hoc one-way ANOVA using the average AUC of each experimental replicate (n=3-4) and the p-values show the comparison of the respective biosensors to Nir1-LNS2 (F = 12.74, P-value < 0.0001, R^2^ = 0.8244). **(K)** Stimulating HEK293A cells with 100 nM PMA and 750 nM of the PLD inhibitor FIPI reduces the Nir1-LNS2 response to PMA. Data shown is the grand mean of 3 experiments ± SEM. A total of 27-41 cells were analyzed.

The most widely used PA biosensors are those that utilize the amphipathic helix of a sporulation-specific soluble *N*-ethylmaleimide sensitive factor attachment protein receptor (SNARE) from *S. cerevisiae*: Spo20p (Figure 1I) (Zeniou-Meyer et al., 2007; Bohdanowicz et al., 2013; Zhang et al., 2014). This amphipathic helix, made of residues 51-91 and subsequently referred to as PABD-Spo20, binds PA *in vitro* and in yeast (Nakanishi et al., 2004). Interestingly, in human cell lines, PABD-Spo20 is highly localized in the nucleus, but it does bind the PM when PA levels are increased (Du and Frohman, 2009; Zeniou-Meyer et al., 2007).

To stop PABD-Spo20 from accumulating in the nucleus, as the small helix is thought to act as a nuclear localization sequence, Zhang et al. (2014) added a nuclear export sequence (NES) to PABD-Spo20, naming this sensor the PA biosensor with superior sensitivity (PASS). PASS shows specificity for PA *in vitro* and dependency on PA for membrane binding in cells (Zhang et al., 2014). An additional PABD was then added to PASS to increase the avidity by enabling the sensor to bind to two PA molecules. We refer to this sensor as NES-PABDx2-Spo20 (Bohdanowicz et al., 2013).

A caveat to these Spo20-based biosensors is that PABD-Spo20 also binds phosphatidylinositol 4,5-bisphosphate (PIP_2_) and phosphatidylinositol 4-phosphate (PI4P) in biochemical assays. It was even suggested to have non-specific interactions with any negatively charged lipids present in the membrane (Nakanishi et al., 2004; Horchani et al., 2014). This highlights the three major problems in the design of PA biosensors: (1) we have yet to discover a sequence or domain structure that is specific for PA binding, (2) amphipathic helices like the PABD tend to indiscriminately interact with membranes, and (3) PA is a negatively charged lipid with a simple structure. Therefore, it can be unclear whether a sensor is specific for PA or whether it has a general affinity for negatively charged membranes.

When looking for a more PA-specific domain to use as a biosensor, we investigated the Nir family of phosphatidylinositol transfer proteins (PITPs). This family of proteins, made up of Nir1, Nir2, and Nir3, form ER-PM membrane contact sites (MCS) to exchange PA and phosphatidylinositol (PI) between the compartments (Cockcroft and Raghu, 2016; Kim et al., 2015). The Nir proteins contain a C-terminal Lipin/Nde1/Smp2 (LNS2) domain (Figure 1I). The AlphaFold predicted structure of this domain shows similarity to the Lipin/Pah family of phosphatidic acid hydrolases (PAPs). Lipin/Pah PAPs interact with the membrane through an N-terminal amphipathic helix and then catalyze the dephosphorylation of PA through a DxDxT-containing Mg^2+^-binding active site (Khayyo et al., 2020). These features are conserved in the Nir LNS2 domains, except for the catalytic Asp in the DxDxT motif and another Mg^2+^-coordinating residue. Therefore, the Nirs are suggested to sense PA levels with their LNS2 domain but not dephosphorylate the lipid (Kim et al., 2013). As the LNS2 tertiary structure is unique compared to the helical nature of PABD-Spo20, we investigated Nir1-LNS2 as a putative novel PABD.

The literature suggests that the Nir family LNS2 domains are specific for PA *in vitro*, and bind the PM in a PA-dependent way, both as an isolated domain and in the context of the full-length Nir proteins (Kim et al., 2013; Chang and Liou, 2015; Quintanilla et al., 2022). However, other studies have suggested that the Nir proteins respond to changes in diacylglycerol (DAG) as well as PA (Kim et al., 2015). Therefore, membrane binding of this domain must be further characterized to determine if it meets the criteria of a valid PA biosensor.

In this study, we set out to corroborate Nir1-LNS2 as a novel and high-affinity PA biosensor. We used pharmacological stimulation of HEK293A cells, liposome binding assays with the purified Nir1-LNS2, and chemically inducible dimerization systems to show that Nir1-LNS2 convincingly reports changes in PA levels at the PM and is more sensitive than Spo20-based biosensors. Furthermore, Nir1-LNS2 exhibits properties of a high-quality PA biosensor: it binds PA and PIP_2_ in liposomes, its membrane interactions are dependent on PA in cells, and PA is sufficient to recruit Nir1-LNS2 to cellular membranes. We then use the Nir1-LNS2 to demonstrate differences in PA production in various cell models, as well as uncover that endogenous PLD activity contributes to PA levels after PLC activation. Thus, this work defines Nir1-LNS2 as a novel tool for the study of PA which reveals aspects of PA regulation that have not been detected by previous biosensors.

## Results

### Nir1-LNS2 is highly sensitive to PA.

Before setting out to design a novel PA biosensor, we first characterized the Spo20 PABD for use as a positive control. We stimulated HEK293A cells expressing the Spo20 biosensors with 100 nM phorbol 12-myrstate 13-acetate (PMA). PMA is a phorbol ester that activates PKC, which then activates PLD, the latter of which hydrolyzes phosphatidylcholine (PC) to produce PA at the PM (Castagna et al., 1982; Liang et al., 2019). To measure the PM localization of biosensors throughout this study, we used fluorescent PM markers: either the PIP_2_ biosensor iRFP-PH- PLCδ1, a PM localized fluorophore utilizing the CAAX motif from HRAS (TagBFP-HRAS-CAAX), or CellMask Deep Red (Várnai and Balla, 1998; Idevall-Hagren et al., 2012). We then calculated the PM/cytoplasmic fluorescence intensity ratio of the biosensors, which will increase as the biosensor translocates to bind PA at the PM (Wills et al., 2021).

First, we used a NeonGreen (NG)-tagged *S. cerevisiae* Spo20 PABD with an added NES (Kim et al., 2015), mimicking the design of PASS (Zhang et al., 2014). This biosensor, named NES-PABD-Spo20, only showed minimal PM localization after 15 minutes of PMA stimulation (Figure 1A). Unexpectedly, this biosensor had strong localization in the nucleus of HEK293A cells. We then tested PASS itself against the NES-PABD-Spo20, and while PASS only showed slightly more PM binding after PMA stimulation, the PASS sensor was strongly excluded from the nucleus (Figure 1B).

We determined that the key difference between NES-PABD-Spo20 and PASS was the location of the NES: in NES-PABD-Spo20 it is N-terminal to the NG, whereas in PASS it is inserted between the C-terminus of NG and the PABD (Figure 1A, 1B). When looking at the structure of NES-PABD-Spo20 using Colabfold (Mirdita et al., 2022), we observed that the linker of the NES-PABD-Spo20 forms an alpha helix at the C-terminus of NG (represented by cylinders in Figure 1). We hypothesized this helix may block nuclear exporters from accessing the NES and that the structure of PASS avoids this hinderance by placing both the PABD and the NES on the C-terminal end of NG (Figure 1B). To test this idea, we re-designed the sensor to replace the helical linker with a flexible Ser/Gly-rich linker, naming this biosensor NES-flex-PABD-Spo20. The flexible linker is sufficient to stop nuclear localization of the sensor, although NES-flex-PABD-Spo20 still only shows slight responsiveness to PMA (Figure 1C). This suggests that placement of the NES within a biosensor’s structure is important for its efficacy. However, regardless of PABD-Spo20’s basal localization, biosensors utilizing this domain are not very sensitive to PA levels at the PM.

To increase the PA binding ability of the PABD-Spo20 biosensors, we replicated the design for a biosensor with tandem PABD domains (NES-PABDx2-Spo20) (Bohdanowicz et al., 2013). NES-PABDx2-Spo20 showed strong nuclear localization in our HEK293A cells, presumably due to steric hinderance by same helical linker. However, the tandem PABDs did increase the response of NES-PABDx2-Spo20 to PMA (Figure 1D).

Finally, to try and make the NES in the tandem biosensor more accessible, we added an additional NES on the N-terminus of the NG, naming this sensor NESx2-PABDx2-Spo20. The addition of this second nuclear export sequence did lower the amount of sensor localized in the nucleus of unstimulated cells. Although NESx2-PABDx2-Spo20 did not bind the PM as well as NES-PABDx2-Spo20 did after PMA stimulation (Figure 1E). It should be noted that of the Spo20 biosensors, NES-PABDx2-Spo20 had a larger response to PMA than PASS did, and is thus the biosensor we use throughout the rest of this study (Figure 1J; Table S1).

To develop a PA biosensor that shows robust PM localization when PA is produced, we examined the LNS2 domain of *H. sapiens* Nir1, defined as residues 613 to 897, which looked promising as a PABD based on its structural prediction in AlphaFold (Uniprot: Q9BZ71) that shared similarities with the Lipin/Pah PAPs.

In comparison to the Spo20-based sensors, NG-tagged Nir1-LNS2 showed an even distribution between the PM, the cytoplasm, and the nucleus in unstimulated HEK293A cells. After PMA stimulation, Nir1-LNS2 noticeably translocated to the PM, more so than any of the Spo20-based biosensors (Figure 1F). This suggests that Nir1-LNS2 could serve as a highly sensitive PA biosensor. We then tested the LNS2 domains of the other two Nir family members, Nir2 and Nir3, to determine their sensitivity to PA. The boundaries of the Nir2-LNS2 (Uniprot: O00562) and Nir3-LNS2 (Uniprot: Q9BZ72) were also defined using AlphaFold predictions of the structure of these domains. However, Nir2-LNS2 and Nir3-LNS2 did not have as strong of a response to PMA as Nir1-LNS2 did (Figure 1G, 1H). When looking at the total area under the curve (AUC) for all the Spo20 and Nir biosensors tested, we observed that all of the sensors responded to PMA significantly less than Nir1-LNS2 did (Figure 1J; Table S1).

Next, we confirmed that the PM binding of Nir1-LNS2 after PMA stimulation was dependent on PLD activation and an increase in PA. To do this, we simultaneously stimulated the cells with PMA and the PLD1/PLD2 inhibitor 5-fluoro-2-indolyl des-chlorohalopemide (FIPI) (Su et al., 2009; Liang et al., 2019). Treatment with FIPI significantly reduced the translocation of Nir1-LNS2 to the PM, demonstrating that PA is necessary for PM localization of Nir1-LNS2 (Figure 1K).

Altogether, this suggests that Nir1-LNS2 is more sensitive to PA production by PLD than the Spo20 biosensors or the LNS2 domains from Nir2 and Nir3. Furthermore, Nir1-LNS2 avoids the strong nuclear localization of the PABD-Spo20 helix. Therefore, we went on to further characterize the membrane binding of this domain to validate its use as a high affinity PA biosensor.

### Nir1-LNS2 shows specificity for PA and PIP_2_ in vitro, based on a novel domain structure.

**Figure 2.**
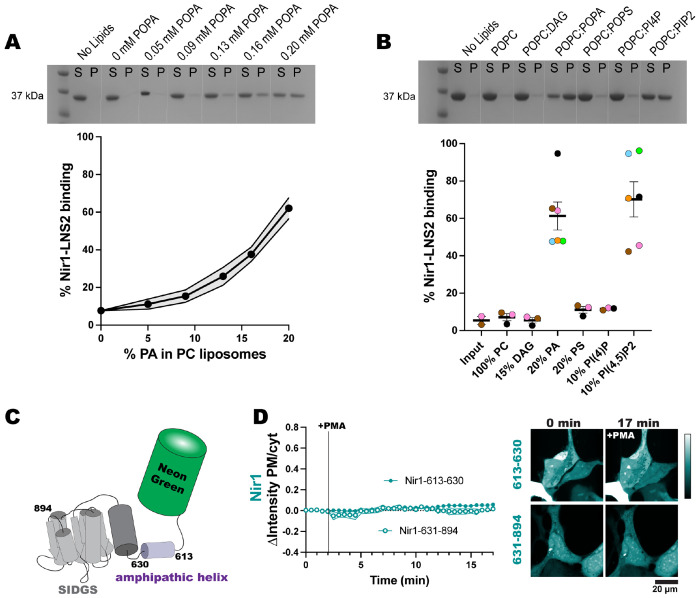
Nir1-LNS2 shows specificity for PA and PIP_2_ *in vitro* based on a novel domain structure. **(A)** A representative SDS-PAGE gel is shown for Nir1-LNS2 binding of increasing PA molar concentrations in POPC liposomes. **(B)** A representative SDS-PAGE gel is shown for Nir1-LNS2 binding to various PM lipids in POPC liposomes. In both A and B, Nir1-LNS2 appears on the gel at 37 kDa. Supernatant (S) and pellet (P) lanes were quantified using ImageJ to determine percent protein bound. The protein-only control pellet was used as a baseline (input). **(C)** Cartoon representing the alpha-fold predicted domain architecture of Nir1-LNS2, which includes an amphipathic alpha helix spanning residues 613-630 and a large, structured domain at residues 631-894, that contains the suspected PA binding motif SIDGS. **(D)** Isolation of either of these domains destroys the ability of Nir1-LNS2 to respond to 100 nM PMA in HEK293A cells. The graph shows the grand means ± SEM of 3-4 experiments (35-42 total cells).

To experimentally probe the lipid-binding specificity of the Nir1-LNS2 domain, we purified a recombinant 6xHis-tagged Nir1-LNS2 protein (residues 604-912) from *E. coli* and performed liposome co-sedimentation to monitor membrane recruitment. Liposomes were made with palmitoyloleoyl (PO) phospholipids to best represent the lipid composition of cellular membranes. In line with prior results, we observed no binding to PC-only liposomes (Kim et al., 2013, 2015; Chang and Liou, 2015). Using this same PC background, we tested the efficacy of other PM lipids in recruiting Nir1-LNS2 to membranes. We found that Nir1-LNS2 bound PA-rich liposomes in a concentration dependent manner (Figure 2A). In addition to binding PA-rich liposomes, Nir1-LNS2 was also specifically recruited to liposomes enriched with PIP_2_, but not to liposomes enriched with DAG or other anionic lipids such as phosphatidylserine (PS) and PI4P (Figure 2B). Overall, this suggests that Nir1-LNS2 binds to both PA and PIP_2_ *in vitro*, but does not generally bind all anionic lipids.

We next determined how Nir1-LNS2 structurally binds to PA. Sequence homology of Nir1-LNS2 together with AlphaFold structure predictions showed a high degree of structural similarity to the Lipin family of enzymes, minus key residues necessary for Mg^2+^ binding and catalysis. The putative Lipin catalytic motif DxDxT is partially conserved in Nir1-LNS2 as a SIDGS motif spanning residues 742-746, suggesting that this SIDGS motif is the assumed PA binding site (Figure 2C) (Kim et al., 2013). However, for efficient catalytic activity, the Lipins also require an N-terminal amphipathic helix for membrane interaction. This helix is made up of residues 1-18 in *Tetrahymena thermophila* Pah2 (Khayyo et al., 2020), and residues 613-630 in the N-terminus of Nir1-LNS2 are predicted to form a similar amphipathic helix (Figure 2C). We therefore tested whether the N-terminal helix of Nir1-LNS2 was necessary for interaction with PA at the PM. We made two truncations of the Nir1-LNS2 construct: Nir1-613-630 is the isolated amphipathic helix, while Nir1-631-894 is the rest of the domain excluding the helix but including the SIDGS motif. Surprisingly, neither truncated construct responded to PMA by binding the PM (Figure 2D). This suggests that the SIDGS motif alone is not the sole PA binding pocket as the LNS2 domain requires both that motif and the amphipathic helix for sustained binding to membrane-embedded PA.

### Polyanionic lipids only slightly affect Nir1-LNS2 binding at the PM.

Because Nir1-LNS2 bound to PIP_2_ in vitro, we investigated if PIP_2_ mediates the interaction of Nir1-LNS2 with the PM in HEK293A cells. To do this, we utilized a chemically inducible dimerization system with phosphatidylinositol phosphate (PIP) phosphatases linked to FK506 binding protein (FKBP) from the mTOR complex and a PM-anchored FKBP rapamycin binding (FRB) domain. In short, cells expressing the system are stimulated with rapamycin. Rapamycin induces dimerization of the FKBP and FRB, thereby acutely localizing the phosphatases at the PM where they degrade PIPs. Then we can determine the effect of the loss of specific PIPs on Nir1-LNS2 membrane binding.

**Figure 3.**
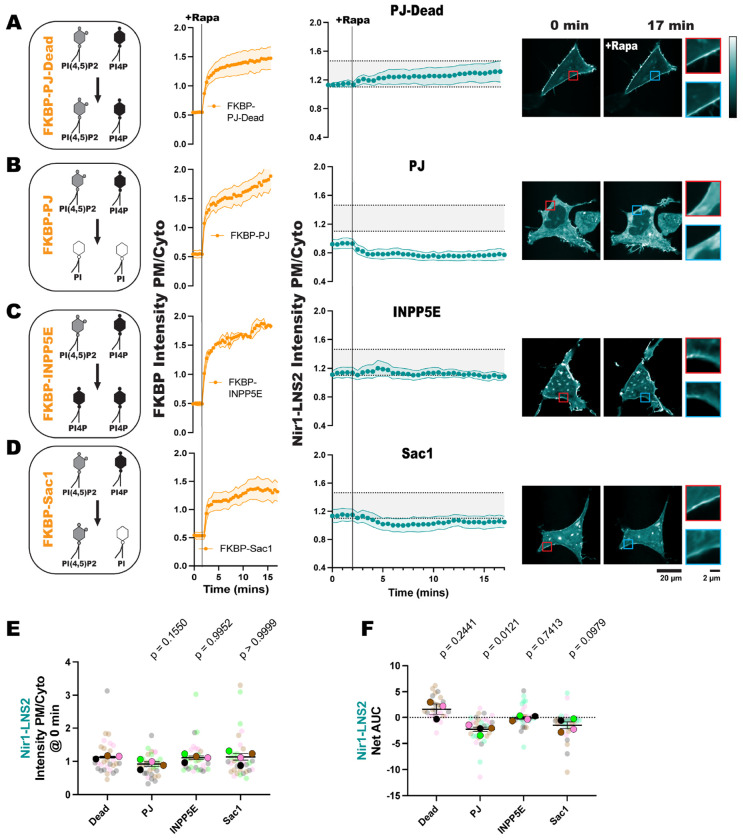
Polyanionic lipids only slightly affect Nir1-LNS2 binding at the PM. FKBP-tagged PIP phosphatases were recruited to the PM by 1 μM rapamycin inducing dimerization between the FKBP fragment and PM-localized FRB. **(A, left)** The FKBP-PJ-Dead does not affect PIP_2_ or PI4P levels, **(A, middle)** despite it robustly recruiting to the PM. **(A, right)** Localization of Nir1-LNS2 was determined during FKBP-PJ-Dead recruitment. In the Nir1-LNS2 graphs, the shaded region indicates the range of this control Nir1-LNS2 response for ease of comparison between conditions. The insets focus on the intensity of Nir1-LNS2 at a region of the membrane before enzyme recruitment (red) and after (blue). **(B)** FKBP-PJ depletes both PIP_2_ and PI4P at the PM. (**C)** FKBP-INPP5E depletes PIP_2_ at the PM but does not reduce PI4P levels. **(D)** FKBP-Sac1 only depletes PI4P at the PM. All xy graphs show the grand means of 3-4 experiments ± SEM. **(E)** The intensity PM/cyto of Nir1-LNS2 before the addition of rapamycin shows the basal activity of the FKBP constructs, which do not significantly alter Nir1-LNS2 localization as compared to the Dead control. Statistics were calculated using a post-hoc one-way ANOVA on the average AUC of each experimental replicate (n=3-4). P-values shown are those compared to the PJ-Dead control (F = 2.220, p. = 0.1431, R^2^ = 0.3771). **(F)** The net area under the curve (AUC) of the Nir1-LNS2 responses was calculated, showing that only PJ slightly reduces Nir1-LNS2’s association with the PM. Statistics were calculated using a one sample t and Wilcoxon test with a hypothetical value of 0 and were computed using the average of each experimental replicate (n=3-4). Dead t = 1.633, df = 2; PJ t = 5.461, df = 3; INPP5E t = 0.3620, df = 3; Sac1 t = 2.377, df = 3. In **(E)** and **(F)**, the small circles indicate values from individual cells (n=29-33). The large circles show the average values for each experimental replicate (n=3-4). Cells in each replicate are color coded accordingly.

To deplete both PIP_2_ and PI4P, we used a chimeric construct Pseudojanin (PJ), consisting of the inositol polyphosphate 5-phosphatase E (INPP5E) and the *S. cerevisiae* Sac1 phosphatase (Hammond et al., 2012). PJ depletes PIP_2_ sequentially, as the INPP5E domain dephosphorylates PIP_2_ to produce PI4P, and then the Sac1 domain dephosphorylates PI4P to produce PI (Figure 3B).

As a negative control, we expressed a doubly catalytically dead mutant of PJ. When PJ-Dead was recruited to the PM, we observed no loss of the PM localization of Nir1-LNS2 (Figure 3A). When the active PJ was expressed in HEK293A cells, there was a slight loss of Nir1-LNS2 at the PM even before PJ recruitment (Figure 3B), although this was not significant as compared to pre-stimulated cells expressing PJ-Dead (Figure 3E). However, Nir1-LNS2 did move off the PM into the cytosol after PJ recruitment (Figure 3B). AUC analysis of the Nir1-LNS2 response showed there was a significant reduction of Nir1-LNS2 PM localization (Figure 3F).

Since PJ depletes both PIP_2_ and PI4P, we examined which of these lipids specifically contribute to Nir1-LNS2 membrane binding. We utilized an FKBP-INPP5E construct that depletes PIP_2_ but does not deplete PI4P at the PM (Figure 3C). Then FKBP-Sac1, an FKBP-PJ construct that has a catalytically dead INPP5E domain, but an active Sac1 domain was used to deplete PI4P without altering PIP_2_ levels (Figure 3D). Recruitment of FKBP-INPP5E did not significantly affect Nir1-LNS2 localization (Figure 3C, 3F). However, recruitment of FKBP-Sac1 slightly, but not significantly affected Nir1-LNS2 localization (Figure 3D, 3F). This data suggests that decreasing the anionic charge of the membrane through depletion of PIPs slightly reduces Nir1-LNS2’s ability to interact with the PM, but it doesn’t fully re-localize the sensor. While this is a small caveat to consider when using Nir1-LNS2 to study PA, the data also demonstrates that Nir1-LNS2 is not specifically interacting with any of the PM PIPs in cellular membranes.

### PA alone is sufficient for Nir1-LNS2 membrane binding.

An often-overlooked criteria for a lipid biosensor is showing that the lipid of interest is sufficient to recruit the sensor to membranes. We saw Nir1-LNS2 bind PIP_2_ *in vitro*, and observed some slight effects on Nir1-LNS2 localization with depletion of PIP_2_ and PI4P at the PM. Therefore, we tested if the PIPs were sufficient for Nir1-LNS2 membrane binding. To do this, we designed a knock sideways system to produce PIPs at the mitochondrial membrane to look at Nir1-LNS2 lipid binding outside the context of the PM (Robinson et al., 2010). The mitochondrial membrane was chosen specifically as it is more isolated from the PM than organelles like the ER or Golgi with which the PM exchanges lipids by vesicular traffic.

**Figure 4.**
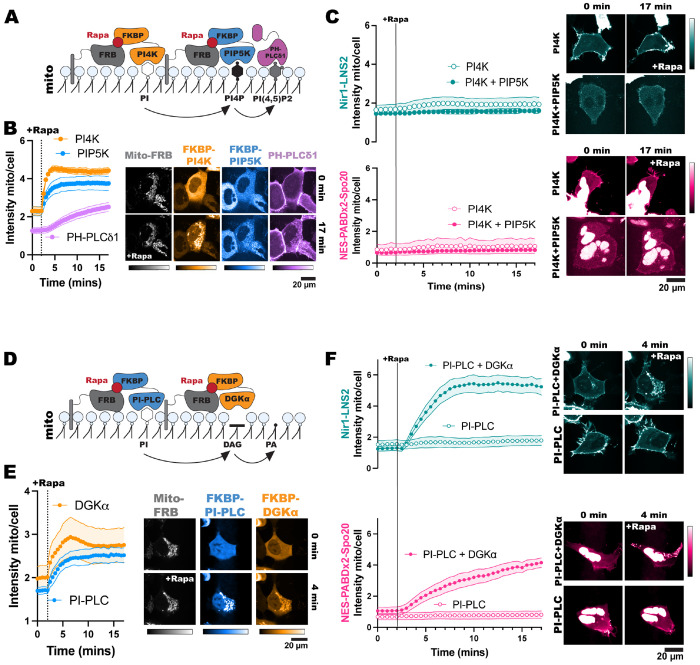
PA alone is sufficient for Nir1-LNS2 membrane binding. **(A)** FKBP-PI4K and FKBP-PIP5K were co-expressed in HEK293A cells to convert PI into PI4P and then PIP_2_ after **(B)** recruitment to mitochondria by the dimerization of the FKBP fragment and mitochondrial-localized FRB with 1 μM rapamycin. The recruitment of the PIP_2_ biosensor PH-PLC*δ*1 to mitochondria shows the efficiency of this system. **(C)** The localization of Nir1-LNS2 and NES-PABDx2-Spo20 was unchanged upon ectopic PI4P and PIP_2_ production. **(D, E)** FKBP-PI-PLC and FKBP-DGKα were co-expressed in cells and recruited to mitochondria to produce DAG from PI, and PA from DAG. **(F)** Nir1-LNS2 and NES-PABDx2-Spo20 were only recruited to mitochondria where PA was produced. All experiments were performed 3-4 independent times, with the xy graphs showing the grand means of the experiments ± SEM. 33-45 total cells were analyzed. Note the Nir1-LNS expressing cell shown in F is the same as shown in panel E.

To make PIPs at the mitochondria, we expressed a mito-FRB in cells. Then we utilized overexpression of an FKBP-tagged phosphatidylinositol 4-kinase (FKBP-PI4K) or co-expression of FKBP-PI4K and an FKBP-tagged phosphatidylinositol 4-phosphate 5-kinase (FKBP-PIP5K). The FKBP-PIP5K was designed by adding point mutations in the full-length *H. sapiens* PIP5K1C (D101R and R304D) that swaps the charge of these positions to stop dimerization with endogenous PIP5Ks (Hu et al., 2015). Additionally, point mutations in the C-terminal domain (R445E and K446E) were used to stop the constitutive PM association of the enzyme such that we could use the FKBP/FRB system to acutely localize it to mitochondria (Suh et al., 2006). At the mitochondria, the FKBP-PI4K converts PI in the membrane into PI4P (Zewe et al., 2020). In cells that co-express the kinases, the PI4P made by FKBP-PI4K is further converted into PIP_2_ by FKBP-PIP5K (Figure 4A).

We validated the efficacy of this system by using PH-PLCδ1 to monitor PIP_2_ production at the mitochondria (Idevall-Hagren et al., 2012; Várnai and Balla, 1998). Rapamycin induced robust recruitment of the FKBP constructs to the mitochondria, which was then followed by PH-PLCδ1 as PIP_2_ was produced (Figure 4B). Upon production of PI4P or PIP_2_ at mitochondria, we did not see any localization of Nir1-LNS2 or NES-PABDx2-Spo20 at this organelle (Figure 4C). Overall, this suggests that PI4P and PIP_2_ are not sufficient to recruit these PA biosensors, and it provides further evidence that Nir1-LNS2 is only engaging in non-specific, electrostatic interactions with these lipids at the PM.

We next used a different mitochondrial chemically inducible dimerization system to produce DAG and PA and test whether these lipids are sufficient for Nir1-LNS2 or NES-PABD-Spo20 membrane binding. We used an FKBP-tagged *B. cereus* phosphatidylinositol PLC (PI-PLC), which uses PI as a substrate to produce DAG (Pemberton et al., 2020). This construct was then co-expressed with an FKBP-tagged catalytic fragment of DGKα to subsequentially produce PA from the DAG (Figure 4D). Both FKBP constructs showed dimerization with mito-FRB leading to mitochondrial localization after rapamycin addition (Figure 4E). However, only cells co-expressing FKBP-PI-PLC and FKBP-DGKα showed any mitochondrial localization of Nir1-LNS2 and NES-PABDx2-Spo20 (Figure 4F). This demonstrates that PA is sufficient to recruit these biosensors to membranes, while DAG is not, thus substantiating that Nir1-LNS2 fulfills this criterion for being a valid PA biosensor.

### The Nir1-LNS2 response to PLC depends on PA and not DAG.

We have shown that Nir1-LNS2 responds to PLD activation by PMA and that PA is sufficient for Nir1-LNS2 membrane binding, so next we investigated whether Nir1-LNS2 is a useful probe to measure PA production downstream of PLC activation. To do this, we used carbachol (CCh) to stimulate the cholinergic receptor muscarinic 3 (CHRM3; referred to as M3) in HEK293A cells and activate PLC. Atropine (Atro) was then used as an antagonist of this receptor to turn off signaling and allow PA levels to return to baseline.

**Figure 5.**
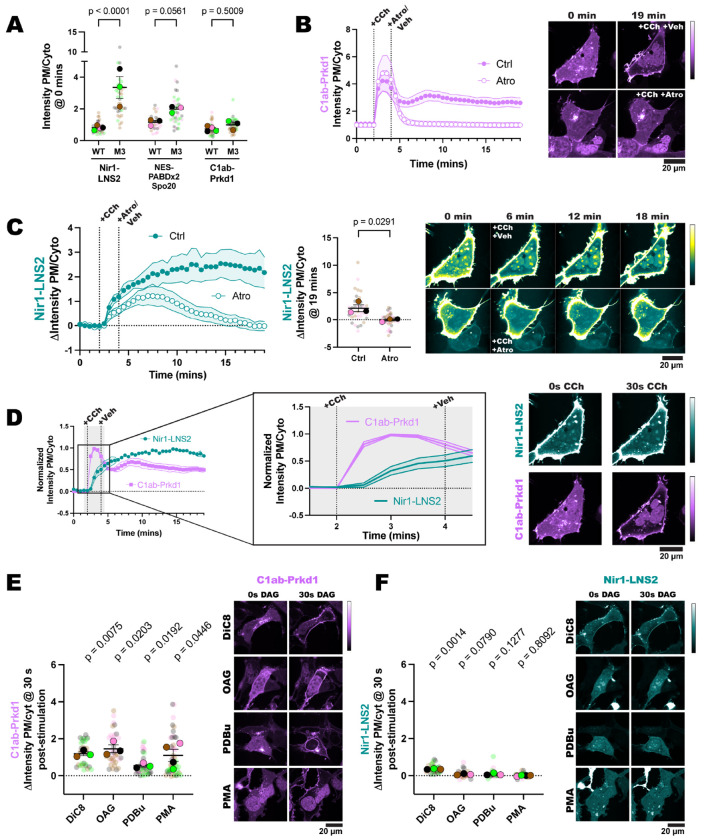
The Nir1-LNS2 response to PLC depends on PA and not DAG. **(A)** Overexpression of the M3 receptor in HEK293A cells elevates Nir1-LNS2 and NES-PABDx2-Spo20 PM localization even without agonist addition. Data was taken from timepoint 0 of experiments in Figures 1C, 1E and 5C. 30-46 cells (small symbols) were analyzed from 3-4 individual experiments (large symbols). Statistics were calculated using an ordinary two-way ANOVA using the average of each experimental replicate (large symbols, n=3-4) DF = 1, MS = 7.185, F(1, 14) = 30.37, p<0.0001. **(B)** The DAG biosensor C1ab-Prkd1 shows the activation and subsequent attenuation of PLC signaling upon addition of 10 μM of the M3 agonist carbachol (CCh) for 2 minutes and then 5 μM of the M3 antagonist atropine for 15 minutes. Control cells were treated with cell media (vehicle) after CCh stimulation. **(C)** Nir1-LNS2 shows increased PM localization after CCh stimulation, but then loss of PM localization upon atropine treatment. Cells were treated as in B. The scatter plot shows the change in intensity ratio of Nir1-LNS2 at the final timepoint. Small symbols represent individual cells (n=38-46) which are color coded according to their experimental replicate shown by the large symbols (n=3-4). Statistics were calculated using an unpaired t-test on the average AUC for each replicate (n=3-4). A two-tailed p-value was used, t = 3.328, df = 4. The PM signal is saturated to see the cytosolic signal, so the images are pseudo-colored to better show Nir1-LNS2 in the cytoplasm as it comes off the PM. **(D)** Nir1-LNS2 translocation to the PM (data replicated from Figure 5C) lags behind C1ab-Prkd1 (data replicated from Figure 5B) in response to CCh addition. The data has been normalized so that the maximum value is 1. All xy graphs show the grand means of each experimental replicate ± SEM. **(E-F)** 30 seconds after HEK293A cells were stimulated with 150 μM DiC8, 150 μM OAG, 5 μM PDBu, or 100 nM PMA, C1ab-Prkd1 translocates to the PM **(E),** but Nir1-LNS2 does not bind the DAG analogs **(F).** The small circles indicate the change in the intensity ratio of individual cells (n=30-46) 30 seconds after stimulation. The large circles show the average change in intensity ratio for each experimental replicate (n=3-4). Cells in each replicate are color coded accordingly. Statistics were calculated using a one sample t and Wilcoxon test with 0 as the hypothetical value. Statistics used the average change in ratio of each experimental replicate (n=3-4). Nir1-LNS2-DiC8: t=26.25, df=2; Nir1-LNS2-OAG: t=3.343, df=2; Nir1-LNS2-PDBu: t=2.523, df=2; Nir1-LNS2-PMA: t=0.2635, df=3. C1ab-Prkd1-DiC8: t=11.49, df=2; C1ab-Prkd1-OAG: t=6.916, df=2; C1ab-Prkd1-PDBu: t=7.118, df=2; C1ab-Prkd1-PMA: t=3.334, df=3. PMA data and the cell shown is duplicated from Figure 1E.

To maximize PLC activation, we overexpressed the M3 receptor in HEK293A cells. Interestingly, we saw that when cells overexpressed this receptor, the basal PM localization of Nir1-LNS2 and NES-PABDx2-Spo20 was significantly elevated compared to wild-type (WT) cells (Figure 5A). This suggests that there is some basal activity of the overexpressed receptors even without exogenous agonist addition. A biosensor made up of the tandem C1 domains of protein kinase D (C1ab-Prkd1), which senses DAG, was used to directly monitor activity of PLC (Kim et al., 2011) However, we did not see any difference in the localization of C1ab-Prkd1 in the M3 overexpression cells versus the WT cells (Figure 5A). We suspect that any increase in DAG is quickly converted to PA by the DGKs, as others have seen DAG clearance around 10 minutes with receptor overexpression (Kim et al., 2015).

We next validated that the addition of atropine was able to halt PLC activity by using the C1ab-Prkd1 biosensor. In control cells treated with CCh for 2 minutes and then vehicle, DAG levels remained elevated after 15 minutes. However, when treated with CCh and then atropine, the DAG levels quickly returned to baseline (Figure 5B). Similarly, Nir1-LNS2 remained at the PM when cells were stimulated with CCh and then vehicle, presumably due to continued elevation of PA. Alternatively, in cells treated with CCh and then atropine, Nir1-LNS2 localized to the PM after CCh was added but was then observed returning to the cytoplasm over the 15-minute treatment with atropine as PA levels declined (Figure 5C). Overall, this experiment shows that Nir1-LNS2 binds the PM reversibly and in a PA-dependent manner.

Since PLC signaling produces DAG, a small lipid that could potentially fit inside the Nir1-LNS2 domain, we wanted to ensure that the observed Nir1-LNS2 response to CCh was not due to DAG production. We normalized the C1ab-Prkd1 and Nir1-LNS2 responses to CCh in the control cells and analyzed the kinetics of each biosensor. When we look at the first 2-minutes after CCh addition, we see that C1ab-Prkd1 moves to the PM much faster than Nir1-LNS2 does (Figure 5D). The delay in Nir1-LNS2 translocation makes sense given DAG is produced first and then converted into PA, again indicating that Nir1-LNS2 is specific for PA.

To further demonstrate that Nir1-LNS2 does not bind to DAG, we stimulated cells with DAG analogs and compared C1ab-Prkd1 and Nir1-LNS2 localization 30 seconds after stimulation. We used 1,2-Dioctanoyl-sn-glycerol (DiC8), 1-oleoyl-2-acetyl-sn-glycerol (OAG), phorbol 12,13-dibutyrate (PDBu), or PMA, which are all analogs of endogenous DAG. As expected, the C1ab-Prkd1 biosensor robustly localized to the PM after 30 sec of stimulation (Figure 5E), since it bound directly to the DAG analogs (Chen et al., 2008). However, none of the DAG analogs caused a large change in the localization of Nir1-LNS2 in this time frame (Figure 5F). We did see some slight PM localization of Nir1-LNS2 after DiC8 stimulation, however these DAG analogs are able to activate PKC and PLD to produce PA (Selvy et al., 2011), which could cause the translocation of Nir1-LNS2 seen. Despite this, the data suggests that Nir1-LNS2 does not bind DAG in cells, which makes it a useful biosensor to help distinguish between the lipid products of PLC signaling.

### Nir1-LNS2 can be used to study endogenous PA signaling in a variety of cell types.

We have confirmed that Nir1-LNS2 is a promising PA biosensor: the purified protein binds PA in artificial membranes, and in cells its membrane interactions depend on PA, and PA is sufficient for its membrane localization. Now, we wanted to demonstrate the applications of Nir1-LNS2 as a high-affinity PA biosensor.

**Figure 6.**
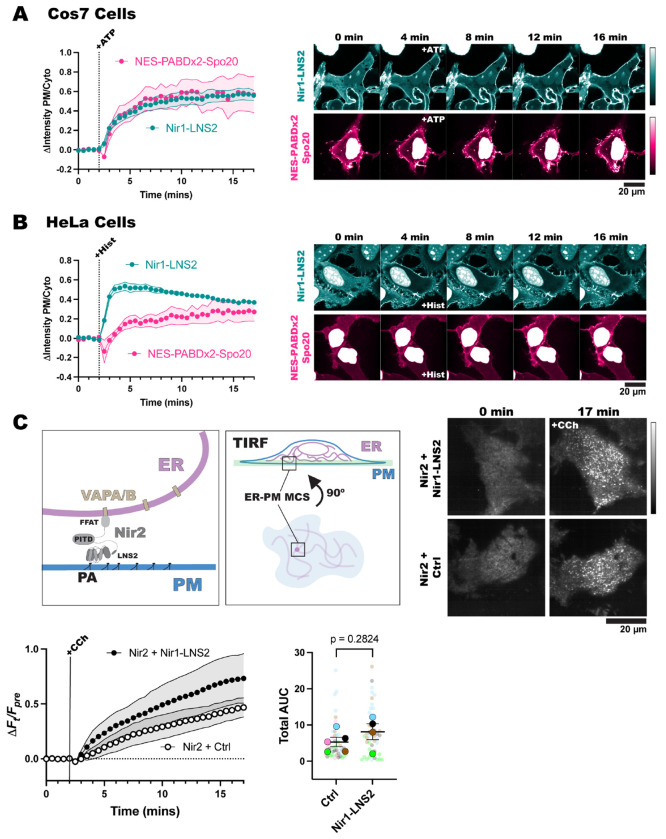
Nir1-LNS2 can be used to study endogenous PA signaling in a variety of cell types. **(A)** Cos7 cells transfected with Nir1-LNS2 and NES-PABDx2-Spo20 show that the biosensors have a similar response to each other when cells are treated with 100 μM ATP. **(B)** HeLa cells transfected with the biosensors show that Nir1-LNS2 responds to treatment with 100 μM histamine more robustly than NES-PABDx2-Spo20. Graphs show grand means ± SEM for 3-4 experiments. A total of 33-57 cells were analyzed. **(C)** Schematic of Nir2 ER-PM MCS formation and how the MCS appear using TIRFM, see text for details. Nir2 was co-expressed with either Nir1-LNS2 or a control biosensor Tubby, which binds PIP_2_. Nir1-LNS2 overexpression did not change the rate that Nir2 formed MCS. The graph shows the grand means ± SEM for 4-5 experimental replicates. Total area under the curve (AUC) for the Nir2 response was plotted. The small circles represent individual cells (n=44-48 cells), and the large circles represent experimental replicates (n=4-5 experiments). Symbols are color coded according to their replicates. Statistics were calculated on the experimental mean of means (n=4-5) with an unpaired t test using a two-tailed p-value. t=1.164, df=7.

First, we validated that Nir1-LNS2 can be utilized in various model cell lines to show PA levels with high affinity. We expressed Nir1-LNS2 and NES-PABDx2-Spo20 in African green monkey kidney cells (Cos7) and HeLa cells. We then stimulated the Cos7 cells with ATP and the HeLa cells with histamine, to activate the cells’ native PLC-coupled purinergic and histamine receptors, respectively. In Cos7 cells, NES-PABDx2-Spo20 responded to ATP just as robustly as Nir1-LNS2 did, which came as a surprise. However, NES-PABDx2-Spo20 still showed strong localization in the nucleus of Cos7 cells, so using Nir1-LNS2 in these cells still offers the advantage of easier visualization of the changes in PA levels (Figure 6A). In HeLa cells, we saw the two biosensors behave similarly to how they did in HEK293A cells. Nir1-LNS2 showed much greater PM binding upon PLC activation, as well as less nuclear localization when compared to NES-PABDx2-Spo20 (Figure 6B). Therefore, we have confirmed that Nir1-LNS2 offers several advantages over NES-PABDx2-Spo20 even in a variety of cell lines.

As Nir1-LNS2 shows high affinity for PA across cell lines, this brings up the concern that use of Nir1-LNS2 will inhibit endogenous signaling pathways that depend on PA. To determine if this is the case, we used Nir2 membrane contact site (MCS) formation as a model of a PA-dependent event. Full-length Nir2 is localized to the ER by interaction of its FFAT motif with the VAPA/B proteins. Then when PA is produced at the PM, the LNS2 of Nir2 binds the PA, bridging the ER and the PM and forming an MCS (Figure 6C) (Cockcroft and Raghu, 2016). We can observe the formation of the MCS using total internal reflection fluorescence microscopy (TIRFM), which selectively excites fluorophores near the bottom of the cell. In TIRFM, Nir2 localized on the ER can be seen as a hazy network and then when Nir2 moves to the PM to form MCS, it appears as bright distinct puncta (Figure 6C). This experiment also avoids artifacts of Nir2 overexpression as only Nir2 interacting with endogenous VAPA/B is able to form MCS.

We co-expressed Nir2 and either Nir1-LNS2 or TubbyC, a PIP_2_ biosensor that is not expected to affect MCS formation. We saw that there was no significant difference in Nir2 MCS formation between these conditions after CCh stimulation (Figure 6C). This suggests that use of Nir1-LNS2 as a PA biosensor does not disrupt endogenous pathways that depend on PA. Therefore, Nir1-LNS2 can be used as a novel, high-affinity biosensor to study a variety of PA signaling pathways without concern of affecting downstream events.

### Nir1-LNS2 reveals that PLD contributes to PA production downstream of PLC.

**Figure 7.**
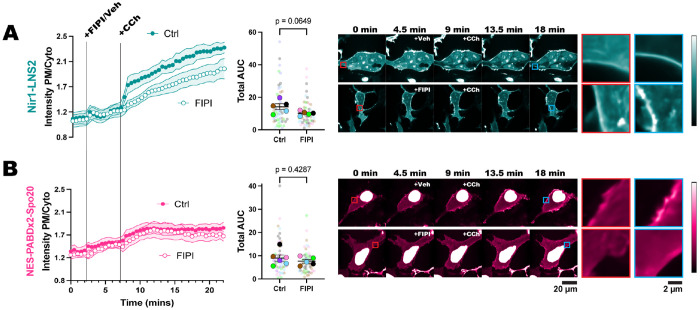
Nir1-LNS2 reveals that PLD contributes to PA production downstream of PLC. HEK293A cells expressing either Nir1-LNS2 **(A)** or NES-PABDx2-Spo20 **(B)** were treated with 750 nM FIPI for 5 minutes to inhibit PLD activity or cell media as a vehicle control, and then 5 μM CCh was added for 15 minutes to induce M3 receptor signaling. The sensitivity of Nir1-LNS2 shows that PLD activity contributes to PA production and the PM localization of Nir1-LNS2. The red insets show the biosensors at a region of the PM before stimulation, while the blue insets show that same region after stimulation. The xy graphs show the grand means of 5-6 experiments ± SEM. The scatter plots show the area under the curve (AUC) of individual cells’ responses as the small symbols (n=48-60) and average AUC of the experimental replicates as the large symbols (n=5-6). Cells are color-coded according to their experimental replicate. Statistics were calculated using a student’s t-test using the average replicate AUC (n=5-6). For Nir1-LNS2, t=2.139, df=8. For NES-PABDx2-Spo20, t=0.8288, df=9. In both tests, a two-tailed p-value was used.

The novelty of Nir1-LNS2 is its high-affinity interaction with PA, and so we hypothesized that this high affinity would allow us to visualize subtle changes in PA levels that cannot be seen with Spo20-based biosensors. Therefore, we utilized Nir1-LNS2 to determine how PA is produced downstream of M3 activation. Stimulation of this receptor with CCh activates PLC and DGK to produce PA but is also thought to activate PKC and PLD (Shulga et al., 2011; Liang et al., 2019). We investigated the specific role of PLD in M3 activation and whether the Nir1-LNS2 biosensor could detect PLD’s contribution to PA levels. To do this, we pre-treated HEK293A cells with the PLD inhibitor FIPI or a vehicle, and then treated with CCh. We see reduced PM localization of the Nir1-LNS2 when cells are treated with FIPI and CCh as compared to vehicle and CCh (Figure 7A). This suggests that PLD is making a small contribution to PA levels downstream of PLC. However, when using NES-PABDx2-Spo20, we do not see any difference in the localization of the sensor in FIPI-treated or vehicle-treated cells (Figure 7B). It should also be noted that Nir1-LNS2 is more responsive to PLC activation by CCh than NES-PABDx2-Spo20 is, just as we saw with PLD activation by PMA. This suggests that the high affinity of Nir1-LNS2 is indeed necessary to deconvolve PLD activity from that of DGK downstream of PLC. Overall, this experiment demonstrates that PA is a lipid with complex regulatory mechanisms that only a high-affinity sensor such as Nir1-LNS2 can untangle. Therefore, we anticipate Nir1-LNS2 will be greatly impact future experiments dissecting PA regulation and the lipid signaling field as a whole.

## Discussion

In this study, we set out to validate the Nir1-LNS2 domain as a novel PA biosensor by characterizing its membrane interactions both *in vitro* and in cells. We saw that Nir1-LNS2 offered several advantages over the current PA biosensors based on the Spo20 PABD. Namely, Nir1-LNS2 has a robust response to PLD activation by PMA, indicating a high-affinity interaction with PA, and Nir1-LNS2 provides clearer confocal images by avoiding heavy nuclear localization (Figure 1). We then characterized Nir1-LNS2 lipid binding using liposomes and determined that there is specificity for both PA and PIP_2_ (Figure 2). However, in live cells, we only saw a slight preference of Nir1-LNS2 for polyanionic lipids, and only PA was sufficient to recruit Nir1-LNS2 to membranes. (Figure 3, Figure 4). Next, we showed that the membrane binding of Nir1-LNS2 depends on the presence of PA (Figure 5). Altogether, these data show that Nir1-LNS2 meets the criteria for a valid biosensor. We then demonstrated that Nir1-LNS2 can be used in a variety of cell types (Figure 6), does not disrupt downstream PA signaling (Figure 6), and importantly, can be used to detect subtle contributions of PLD to the pool of PA that have been difficult to image with previous tools (Figure 7). Overall, we have characterized Nir1-LNS2 as a novel and high-affinity PA biosensor that can be applicable for diverse studies of the PA pathway.

The one caveat when using the Nir1-LNS2 biosensor is the discrepancy in its specificity: *in vitro* it was shown to bind to PA and PIP_2_ (Figure 2), while *in vivo* it solely binds to PA (Figure 4). One reason for this difference could be that the Nir1-LNS2 is not a novel *bona fide* PA binding pocket. Rather, it requires both the SIDGS motif and an N-terminal amphipathic helix for membrane interactions (Figure 2). As the amphipathic helix will generally interact with negatively charged lipids, the helix may play more of a role in membrane interactions when more negative charges are present (Kim et al., 2013; Khayyo et al., 2020). The charges of phospholipids and therefore their ability to recruit the N-terminal helix of Nir1-LNS2 depend on the deprotonation state of the lipid headgroup. In PIP_2_, one of the phosphate groups loses both its protons, and the other only loses one proton at physiological pH (Kooijman et al., 2009). However, the remaining proton forms intermolecular and intramolecular hydrogen bonds with the other phosphates and hydroxyls on lipid headgroups (Kooijman et al., 2009). The presence of other lipids such as phosphatidylethanolamine (PE) or PI, the formation of PIP_2_-rich domains, and even interactions with neighboring proteins can increase hydrogen bonding of PIP_2_ and dilute the negative charge (Graber et al., 2012; Borges-Araújo and Fernandes, 2020). Therefore, we suspect that in cells, the N-terminal helix of Nir1-LNS2 does not interact with the PIP_2_ as strongly due to this spreading of the negative charge. Whereas the PIP_2_ in liposomes is more isolated and therefore has increased charge and more interaction with the N-terminal helix.

Differences in biosensor specificity *in vitro* and *in vivo* have been seen for other biosensors as well. For example, the PH domains of OSBP and FAPP1 bound to liposomes in a PI4P and PIP_2_ dependent manner (Levine and Munro, 2002). However, membrane interactions of these probes in yeast were only dependent on PI4P production (Levine and Munro, 2002). Even with these discrepancies, the biosensors have still proved to be useful in studies of PI4P-dependant processes (Szentpetery et al., 2010). Similarly, we believe that the Nir1-LNS2 will serve as a valid PA biosensor for future studies in live cells.

Despite this drawback in the specificity of Nir1-LNS2, its major use as a PA biosensor stems from its highly sensitive membrane recruitment by PA. The widely used Spo20 biosensors have lower responsiveness to PA production as compared to Nir1-LNS2 (Figure 1), and the high affinity of Nir1-LNS2 allows us to now more easily sense subtle changes in the pool of PA (Figure 7). Previous studies have used PABD-Spo20 to successfully show the specific activity of PLD1 during exocytosis by using PLD1 siRNA (Zeniou-Meyer et al., 2007). Other groups have used FIPI to look the effect of PLD on basal PA levels. Although the authors saw that the effects of FIPI on NES-PABDx2-Spo20 varied depending on the cell type used (Bohdanowicz et al., 2013).

However, looking at PLD activation specifically downstream of muscarinic receptors has remained difficult until a recent study used click chemistry to repurpose the transphosphatidylation reaction catalyzed by PLD to create clickable lipids that can incorporate fluorescent reporters, a technique referred to as real-time IMPACT (Liang et al., 2019). This study corroborated our results, showing that PLD is activated at the PM by stimulation of the muscarinic M1 receptor. While real-time IMPACT does not directly report on PA levels as it does not use the endogenous PLD substrate PC, it offers several advantages such as being able to interrogate lipid trafficking over time. Since the resulting phosphatidyl alcohols are not rapidly metabolized via the same pathways as PA, the fate of these lipids can be continuously monitored. Thus, these PLD-produced fluorescent lipids were determined to traffic from the PM to the ER with a half-life of around 104 seconds, which we were not able to directly observe using Nir1-LNS2 or the Spo20-based biosensors. However, we did see Nir2 MCS formation occur in cells expressing the biosensors (Figure 6), which is thought to mediate PA trafficking to the ER (Kim et al., 2015), so we believe trafficking to still be occurring even if the Nir1-LNS2 probe cannot be used to directly visualize it.

Overall, we want to emphasize that Nir1-LNS2 should not replace current tools such as real-time IMPACT or Spo20 biosensors such as PASS. In this work, we have further characterized the Spo20 PABD by demonstrating that PA is sufficient to recruit NES-PABDx2-Spo20 to membranes (Figure 4). Therefore, our data support Spo20 biosensors as valid and robustly characterized options for low-affinity PA biosensors. There are various situations where it is particularly useful to have both a low-affinity and high-affinity lipid biosensor. For instance, high-affinity biosensors aren’t very effective at quantifying increases in a lipid as the sensor can already be saturated on the membrane. Conversely, low-affinity biosensors struggle to show decreases in a lipid since there is already so much noise in the cytosol (Wills et al., 2018). A recent paper demonstrated the usefulness of having multiple biosensors, describing both a high-affinity cholesterol biosensor, GRAM-W, and its low-affinity counterpart, GRAM-H. The authors used GRAM-W and GRAM-H in parallel to successfully detect decreases and increases in accessible cholesterol (Koh et al., 2023). Therefore, we introduce Nir1-LNS2 as a novel tool in the study of PA to be used in combination with existing tools to aid our understanding of this important lipid.

## Materials and Methods

### Protein Overexpression and Purification.

The full-length Nir1 gene (accession code: NC_000017.11) was codon optimized for expression in *E. coli* and gene synthesized (Twist Bioscience) in the pET28 plasmid. DNA oligo primers were synthesized (Integrated DNA Technologies) for Nir1-LNS2 using residues 609-912 and inserted into the pTHT vector, a modified pET-28 plasmid containing a TEV-cleavable N-terminal 6xHis-tag. The construct was verified with direct sequencing.

The Nir1-LNS2 plasmid was transformed into competent BL21 (DE3) RIPL cells (Agilent Technologies, Cat. No. 230280) for protein overexpression. Cells were grown at 37 °C to an OD600 of 1.5 and induced with 100 μM isopropyl β-D-1-thiogalactopyranoside (IPTG) at 15 °C for overnight growth. Cell pellets were harvested and lysed via sonication in buffer comprised of 50 mM Tris pH 7.5, 500 mM NaCl, 5% glycerol, 1% Triton X-100, and 2 mM beta-mercaptoethanol (βME), and lysates were centrifuged at 82,000 x g at 4 °C for 1 hr. Protein-rich supernatant was collected, and the affinity-tagged proteins were isolated using Ni-NTA gravity flow affinity chromatography and eluted with buffer comprised of 50 mM Tris pH 7.5, 500 mM NaCl, 300 mM imidazole pH 7.5, and 5 mM βME. Isolated proteins were applied to a Superdex 75 26/60 HiLoad column (GE Healthcare) equilibrated with buffer comprised of 20 mM Tris pH 7.5, 150 mM NaCl, 10 mM βME, and 1 mM dithiothreitol (DTT). The purified protein was concentrated to 1-5 mg/mL, flash frozen in liquid nitrogen in 30 βL aliquots, and stored at −80 °C.

### Liposome Co-sedimentation.

Palmitoyl oleoyl (PO) phospholipids (Avanti Polar Lipids) dissolved in chloroform:methanol solution were dried under nitrogen gas and resuspended in Buffer A comprised of 150 mM NaCl and 20 mM Tris pH 7.5 to generate a 2 mM solubilized lipid mixture. Solubilized PO lipids underwent five freeze/thaw cycles with liquid nitrogen and were subsequently sonicated for two minutes in a water bath. 50 μL of pure Nir1-LNS2 at ~1 mg/mL was incubated with 50 μL of liposome mixture for 30 minutes at room temperature. Reactions were then centrifuged in a vacuum for 1 hr at 100,000 g at 4 °C. 75 μL of the supernatant (S) was collected and the liposome pellet (P) was resuspended in 100 μL of buffer A and 75 μL was collected for samples to be resolved via SDS-PAGE. ImageJ software was used to quantify pixel intensity of the S and P fraction gel bands for each condition, and percent protein bound was found using [P/(P+S)]*100.

### Cell Culture and Transfection

HEK293A cells (Invitrogen R70507), Cos7 cells (ATCC CRL-1651), and HeLa cells (ATCC CCL-2) were maintained in complete DMEM comprised of low glucose DMEM (Life Technologies 10567022), 10% heat-inactivated fetal bovine serum (Life Technologies 10438-034), 1% 10,000 units/mL streptomycin + penicillin (Life Technologies 15140122), and 0.1% chemically defined lipid supplement (Life Technologies 11905031). Cells were grown at 37° C and 5% CO_2_. The line was passaged with a 1:5 dilution twice per week after rinsing with PBS and dissociating in TrpLE (Life Technologies 12604039).

For imaging, cells were seeded onto coated 35 mm glass bottom dishes with a 20 mm glass aperture (Fisher Scientific D35-20-1.5-N). HEK293A cells were seeded onto dishes that had been coated with 10 *μ*g ECL cell attachment matrix (Sigma 08-110) in DMEM or Stem Cell Qualified ECM gel (Sigma-Aldrich CC131) diluted 1:80 in DMEM. HeLa and COS-7 cells were seeded onto dishes coated with 5 mg fibronectin (Life Technologies 33016-015) in diH2O. The volume of cells seeded was calculated so that cells would be 90-100% confluent on the day of confocal imaging and 40-50% confluent on the day of TIRF imaging.

After allowing cells to adhere and spread on the dish for 2+ hours, plasmids were transfected into the cells using Lipofectamine2000 (Life Technologies 11668019). 1 *μ*g of DNA was complexed with 3 *μ*g of Lipofectamine2000. This mixture was diluted to 200 *μ*L in Opti-MEM (Life Technologies 51985091) and incubated together for 5 minutes up to 2 hours before being added to the cells. HEK293A and HeLa cells were treated with the DNA and Lipofectamine solution for 3-4 hours before the solution was removed and the cells were placed in 1.6-2 mL of imaging media for imaging the next day. For Cos7 cells, the DNA and Lipofectamine solution was left on the cells for 12-16 hours before being replaced with the appropriate imaging media.

The imaging media, complete HEPES-buffered imaging media (CHIM), was made of Fluorobrite media (Life Technologies A1896702), 10% heat-inactivated fetal bovine serum, 1% Glutamax (Life Technologies 35050061), 25 mM Na-HEPES pH 7.4 (VWR EM-5320), and 0.1% chemically defined lipid supplement. In some experiments, serum-free CHIM + 0.1% BSA (SF-CHIM + 0.1% BSA) was used for imaging. This media was made using the same recipe as CHIM, excluding the HI-FBS and supplementing with 0.1% BSA (Life Technologies 15260-037). CHIM was used for the experiments in Figures 4, 5A–5D, and 6 while SF-CHIM + 0.1% BSA was used in Figures 1, 2, 3, 5E–5F, and 7.

### Confocal Microscopy

The transfected cells were imaged on a Nikon A1R-HD resonant laser scanning confocal microscope, using an inverted TiE microscope stand. Resonant mode was used with a 100x 1.45 NA plan apochromatic oil immersion objective. A dual fiber-coupled LUN-V laser launch was used to excite fluorophores. One line scan used 488 and 640 nm lasers to co-excite green (NG or EGFP) and far red (iRFP) fluorescence. A second line scan used 561 and 405 nm lasers to co-excite red (mCherry or mRFP) and blue (BFP) fluorescence to avoid crosstalk. Emission was collected using individual filters for blue (425-475 nm), green (500-550 nm), yellow/orange (570-620 nm), and far-red (663-737 nm). The pinhole used was calculated to be 1.2x the Airy disc size of the longest wave-length channel used in the experiment. To decrease noise in the images, 8x frame averages were taken, and in some experiments Nikon Elements denoising software was used.

To stain the PM with CellMask Deep Red (Life Technologies C10046), a 2.5 ng/*μ*L solution was made up in the appropriate cell imaging media. Cells were incubated with 500 *μ*L of the CellMask solution for 5 minutes. The cells were then washed with imaging media and 1.6-2 mL of imaging media was added for imaging.

Time-lapse imaging was performed over 17-32 mins, as indicated in the figure legends. 10-15 fields of cells were selected and imaged every 30 sec. Stimulations were added after the time point indicated in the figure legends. Cell stimulations were created by diluting the reagents in the appropriate imaging media as outlined in Table 1. The stimulations were made at a 5x concentration, and then 500 *μ*L of stimulation was added to the 2 mL of imaging media in the dish to produce the final concentration described in Table 1. For experiments that used two consecutive stimulations, cells were imaged in 1.6 mL media and 400 *μ*L of the first stimulation was added and then 500 *μ*L of the second stimulation was added.

### Confocal Image Quantification

Confocal image analysis was done using FIJI and custom macros (Schindelin et al., 2012). Images were imported into FIJI and then displayed as a montage of all xy positions for each specific channel. ROIs were then drawn in the background, around the cell, and in some experiments, within the cytosol of each cell. Cells that moved too much during imaging were excluded from analysis.

We quantified the signal of constructs at the PM or the mitochondria by using a PM or mitochondria marker to create a binary mask at the relevant organelle. The PM was marked by either iRFP-PH-PLCδ1, BFP-HRAS-CAAX, PM-FRB or CellMask deep red. The mitochondria were marked by Mito-FRB. The resulting mask was then used to measure the normalized intensity of a given construct across the time lapse at these membranes. Then, the intensity of the construct within the mask was divided by the intensity within either a cytoplasmic or whole-cell ROI to create the reported ratios.

To create these masks, the PM and mitochondria marker images were filtered with a Gaussian blur filter at 1x, 2x, 3x, and 4x the airy disc size of the marker fluorophore. Wavelets were then generated by subtracting each filtered image from the image filtered at the next smaller length scale. To create the binary mask, the wavelets were multiplied and a threshold of 0.5x standard deviations of the original image was applied. The mask then underwent a 2-pixel dilation cycle to ensure that the whole area of the relevant organelle was included. Further details on this analysis protocol can be found in Wills et al., 2021.

### Total Internal Reflection Fluorescence Microscopy (TIRFM)

Transfected cells were imaged on a Nikon motorized TIRF illuminator mounted on a Nikon TiE inverted microscope stand, using a 100x 1.45 NA plan-apochromatic objective. An Oxxius L4C laser launch was used to excite the following fluorophores: 488-nm for EGFP/NG, 561-nm for mCherry, and 638-nm for iRFP. Single pass chroma filters were used to collect yellow/orange (570-620 nm) and green (505-550 nm) emission, and a dual pass green/far-red filter was used to collect far-red emission (650-850 nm). To image the time lapse, 10-15 individual fields were marked and imaged every 30 s using an Andor Zyla 5.5 sCMOS camera. The fields were imaged using an exposure time of 50 ms and 2x2 pixel binning.

Stimulations were added after 2 minutes of baseline imaging, as indicated in the figure legends. Cell stimulations were created by diluting the reagents in the appropriate imaging media as outlined in Table 1. A 5x solution of the stimulant in cell media was made, and then 500 *μ*L of stimulation was added to the 2 mL of imaging media in the dish to produce the final concentration described in Table 1.

### TIRFM Image Quantification

TIRFM image analysis was done using FIJI and custom-written macros (Schindelin et al., 2012). Images were imported into FIJI and then displayed as montages of each position for each channel. ROIs around the cell footprint were drawn using a minimum intensity projection to account for any movement of the cell during imaging. However, cells were excluded from analysis if they had movement that was too large. An additional ROI was drawn in the background of each field.

The background fluorescence was then subtracted from each field, and the normalized intensity within the cell ROI was measured at each timepoint (F_t_) and normalized to the intensity within the first 5 frames that were taken before stimulation (F_pre_).

### Data Presentation and Statistics

Data analysis, statistics and graphs were done using Graphpad Prism 9. Statistical tests used are provided in the figure legends.

### Plasmids and Cloning

The plasmids used in this study were obtained from the sources as noted in Table 2 or made by either restriction digest and ligation or PCR and NEBuilder HiFi DNA assembly (New England Biolabs E5520S). Insert sequences were ordered as custom GeneBlocks from IDT or isolated from existing plasmids. All plasmid sequences were verified over the relevant area by Sanger sequencing or over the full plasmid with long-read nanopore sequencing. Plasmids created in this study are available on Addgene.

## Supplementary Material

1

## Figures and Tables

**Table 1.** Reagents used for cell stimulation throughout this study.

Reagent	Manufacturer	Catalog Number	Stock Solution	Storage Temperature	Concentration Added to Cells (diluted in cell media)
PMA	Millipore Sigma	P8139	437 *μ*M in DMSO	−20° C	100 nM
FIPI	Millipore Sigma	528245	750 *μ*M in DMSO	−20° C	750 nM
DiC8	EMD Millipore	317505	30 mM in methanol, dried. Resuspended in 50 mL methanol before use.	−80° C	150 *μ*M
OAG	EMD Millipore	495414	30 mM in methanol, dried. Resuspended in 20 mL DMSO before use.	−80° C	150 *μ*M
PDBu	Sigma	P1269	10 mM in DMSO	−80° C	5 *μ*M
Rapamycin	Sigma	553210	1 mM in DMSO	−20° C	1 *μ*M
Carbachol	Fisher	AC10824	50 mM in dH2O	−20° C	5 *μ*M, 10 *μ*M, or 100 μM
Atropine	Sigma	A0257	25 mM in dH2O	4° C	5 *μ*M
ATP	Sigma	10127523001	100 mM in 100 mM MgCl2 + 200 mM Tris base	−20° C	100 *μ*M
Histamine	Fisher	AC15062	100 mM in dH2O	−20° C	100 *μ*M

**Table 2.** Plasmids used in this study. All genes are *Homo sapiens* unless indicated otherwise. The relevant amino acid residues are noted and colored accordingly. Mutations are described by the position of the residues in the full-length protein and are highlighted in the sequence. Gray amino acid sequences indicate linkers.

Shorthand Name	Full Name	Reference	Sequence
**NES-PABD-Spo20**	*X.leavis* map2k1.L^32-44^-EGFP-*S. cerevisiae* Spo20p^51-91^	(Zeniou-Meyer et al., 2007; Zhang et al., 2014)	MALQKKLEELELDEAPVATMVSKGEELFTGVVPILVELDGDVNGHK FSVSGEGEGDATYGKLTLKFICTTGKLPVPWPTLVTTLTYGVQCFS RYPDHMKQHDFFKSAMPEGYVQERTIFFKDDGNYKTRAEVKFEG DTLVNRIELKGIDFKEDGNILGHKLEYNYNSHNVYIMADKQKNGIKV NFKIRHNIEDGSVQLADHYQQNTPIGDGPVLLPDNHYLSTQSALSK DPNEKRDHMVLLEFVTAAGITLGMDELYKGLRSRASIMDNCSGSR RRDRLHVKLKSLRNKIHKQLHPNCRFDDATKTS*
**PASS**	EGFP-M. musculus PKIa^34-51^-*S. cerevisiae* Spo20p^51-91^	(Zhang et al., 2014; Zeniou-Meyer et al., 2007)	MVSKGEELFTGVVPILVELDGDVNGHKFSVSGEGEGDATYGKLTL KFICTTGKLPVPWPTLVTTLTYGVQCFSRYPDHMKQHDFFKSAMP EGYVQERTIFFKDDGNYKTRAEVKFEGDTLVNRIELKGIDFKEDGNI LGHKLEYNYNSHNVYIMADKQKNGIKVNFKIRHNIEDGSVQLADHY QQNTPIGDGPVLLPDNHYLSTQSKLSKDPNEKRDHMVLLEFVTAA GITLGMDELYKSGLRSRANSNELALKLAGLDINKTESRMDNCSASR RRDRLHVKLKSLRNKIHKQLHPNCRFDDATKTS*
**NES-flex-PABD-Spo20**	*X.leavis* map2k1.L^32-44^-EGFP-SGGGSGGS-*S. cerevisiae* Spo20p^51-91^	This Study	MALQKKLEELELDEAPVATMVSKGEELFTGVVPILVELDGDVNGHK FSVSGEGEGDATYGKLTLKFICTTGKLPVPWPTLVTTLTYGVQCFS RYPDHMKQHDFFKSAMPEGYVQERTIFFKDDGNYKTRAEVKFEG DTLVNRIELKGIDFKEDGNILGHKLEYNYNSHNVYIMADKQKNGIKV NFKIRHNIEDGSVQLADHYQQNTPIGDGPVLLPDNHYLSTQSALSK DPNEKRDHMVLLEFVTAAGITLGMDELYKSGGGSGGSMDNCSGS RRRDRLHVKLKSLRNKIHKQLHPNCRFDDATKTS*
**NES-PABDx2-Spo20**	*X.leavis* map2k1.L^32-44^-EGFP-*S. cerevisiae* Spo20p^51-91^- *S. cerevisiae* Spo20p^51-91^	(Bohdanowicz et al., 2013)	MALQKKLEELELDEAPVATMVSKGEELFTGVVPILVELDGDVNGHK FSVSGEGEGDATYGKLTLKFICTTGKLPVPWPTLVTTLTYGVQCFS RYPDHMKQHDFFKSAMPEGYVQERTIFFKDDGNYKTRAEVKFEG DTLVNRIELKGIDFKEDGNILGHKLEYNYNSHNVYIMADKQKNGIKV NFKIRHNIEDGSVQLADHYQQNTPIGDGPVLLPDNHYLSTQSALSK DPNEKRDHMVLLEFVTAAGITLGMDELYKSGLRSRAMDNCSGSRR RDRLHVKLKSLRNKIHKQLHPNCRFDDATKTSMDNCSGSRRRDRL HVKLKSLRNKIHKQLHPNCRFDDATKTS*
**NESx2-PABDx2-Spo20**	*X.leavis* map2k1.L^32-44^-*X.leavis* map2k1.L^32-44^-EGFP-*S. cerevisiae* Spo20p^51-91^- *S. cerevisiae* Spo20p^51-91^	This Study	MALQKKLEELELDEAGGSGMALQKKLEELELDEAPVATMVSKGEE LFTGVVPILVELDGDVNGHKFSVSGEGEGDATYGKLTLKFICTTGKL PVPWPTLVTTLTYGVQCFSRYPDHMKQHDFFKSAMPEGYVQERTI FFKDDGNYKTRAEVKFEGDTLVNRIELKGIDFKEDGNILGHKLEYNY NSHNVYIMADKQKNGIKVNFKIRHNIEDGSVQLADHYQQNTPIGDG PVLLPDNHYLSTQSALSKDPNEKRDHMVLLEFVTAAGITLGMDELY KSGLRSRAMDNCSGSRRRDRLHVKLKSLRNKIHKQLHPNCRFDDA TKTSMDNCSGSRRRDRLHVKLKSLRNKIHKQLHPNCRFDDATKTS*
**Nir1-LNS2**	NeonGreen-Nir1^613-897^	This Study	MVSKGEEDNMASLPATHELHIFGSINGVDFDMVGQGTGNPNDGY EELNLKSTKGDLQFSPWILVPHIGYGFHQYLPYPDGMSPFQAAMV DGSGYQVHRTMQFEDGASLTVNYRYTYEGSHIKGEAQVKGTGFP ADGPVMTNSLTAADWCRSKKTYPNDKTIISTFKWSYTTGNGKRYR STARTTYTFAKPMAANYLKNQPMYVFRKTELKHSKTELNFKEWQK AFTDVMGMDELYKGGSGGMPANPREKWLRKRTQVKLRNVTANH RANDVIAAEDGPQVLVGRFMYGPLDMVALTGEKVDILVMAEPSSG RWVHLDTEITNSSGRITYNVPRPRRLGVGVYPVKMVVRGDQTCAM SYLTVLPRGMECVVFSIDGSFAASVSIMGSDPKVRPGAVDVVRHW QDLGYMILYITGRPDMQKQRVVSWLSQHNFPQGMIFFSDGLVHDP LRQKAIFLRNLMQECFIKISAAYGSTKDISVYSVLGLPASQIFIVGRPT KKYQTQCQFLSEGYAAHLAALEASHRSRPKKN*
**Nir2-LNS2**	NeonGreen-Nir2^896-1216^	This Study	MVSKGEEDNMASLPATHELHIFGSINGVDFDMVGQGTGNPNDGY EELNLKSTKGDLQFSPWILVPHIGYGFHQYLPYPDGMSPFQAAMV DGSGYQVHRTMQFEDGASLTVNYRYTYEGSHIKGEAQVKGTGFP ADGPVMTNSLTAADWCRSKKTYPNDKTIISTFKWSYTTGNGKRYR STARTTYTFAKPMAANYLKNQPMYVFRKTELKHSKTELNFKEWQK AFTDVMGMDELYKGGSGGMSPAFPREKWQRKRTQVKIRNVTSNH RASDTVVCEGRPQVLSGRFMYGPLDVVTLTGEKVDVYIMTQPLSG KWIHFGTEVTNSSGRLTFPVPPERALGIGVYPVRMVVRGDHTYAE CCLTVVARGTEAVVFSIDGSFTASVSIMGSDPKVRAGAVDVVRHW QDSGYLIVYVTGRPDMQKHRVVAWLSQHNFPHGVVSFCDGLTHD PLRQKAMFLQSLVQEVELNIVAGYGSPKDVAVYAALGLSPSQTYIV GRAVRKLQAQCQFLSDGYVAHLGQLEAGSHSHASSGPPRAALGK SSYGVAAPVDFLRKQSQLLRSRGPSQA*
**Nir3-LNS2**	mEGFP-Nir3^925-1209^	This Study	MVSKGEELFTGVVPILVELDGDVNGHKFSVSGEGEGDATYGKLTL KFICTTGKLPVPWPTLVTTLTYGVQCFSRYPDHMKQHDFFKSAMP EGYVQERTIFFKDDGNYKTRAEVKFEGDTLVNRIELKGIDFKEDGNI LGHKLEYNYNSHNVYIMADKQKNGIKVNFKIRHNIEDGSVQLADHY QQNTPIGDGPVLLPDNHYLSTQSKLSKDPNEKRDHMVLLEFVTAA GITLGMDELYKGGGGSHMPSKPREKWQRKRTHVKLRNVTANHRI NDALANEDGPQVLTGRFMYGPLDMVTLTGEKVDVHIMTQPPSGE WLYLDTLVTNNSGRVSYTIPESHRLGVGVYPIKMVVRGDHTFADSY ITVLPKGTEFVVFSIDGSFAASVSIMGSDPKVRAGAVDVVRHWQDL GYLIIYVTGRPDMQKQRVVAWLAQHNFPHGVVSFCDGLVHDPLRH KANFLKLLISELHLRVHAAYGSTKDVAVYSAISLSPMQIYIVGRPTKK LQQQCQFITDGYAAHLAQLKYSHRARPARN*
**Nir1-613-630**	Nir1^613-630^-NeonGreen	This Study	MPANPREKWLRKRTQVKLRGGSGGMVSKGEEDNMASLPATHEL HIFGSINGVDFDMVGQGTGNPNDGYEELNLKSTKGDLQFSPWILV PHIGYGFHQYLPYPDGMSPFQAAMVDGSGYQVHRTMQFEDGASL TVNYRYTYEGSHIKGEAQVKGTGFPADGPVMTNSLTAADWCRSK KTYPNDKTIISTFKWSYTTGNGKRYRSTARTTYTFAKPMAANYLKN QPMYVFRKTELKHSKTELNFKEWQKAFTDVMGMDELYK*
**Nir1-631-894**	Nir1^631-894^-NeonGreen	This Study	MVSKGEEDNMASLPATHELHIFGSINGVDFDMVGQGTGNPNDGY EELNLKSTKGDLQFSPWILVPHIGYGFHQYLPYPDGMSPFQAAMV DGSGYQVHRTMQFEDGASLTVNYRYTYEGSHIKGEAQVKGTGFP ADGPVMTNSLTAADWCRSKKTYPNDKTIISTFKWSYTTGNGKRYR STARTTYTFAKPMAANYLKNQPMYVFRKTELKHSKTELNFKEWQK AFTDVMGMDELYKGGSGGNVTANHRANDVIAAEDGPQVLVGRFM YGPLDMVALTGEKVDILVMAEPSSGRWVHLDTEITNSSGRITYNVP RPRRLGVGVYPVKMVVRGDQTCAMSYLTVLPRGMECVVFSIDGS FAASVSIMGSDPKVRPGAVDVVRHWQDLGYMILYITGRPDMQKQR VVSWLSQHNFPQGMIFFSDGLVHDPLRQKAIFLRNLMQECFIKISA AYGSTKDISVYSVLGLPASQIFIVGRPTKKYQTQCQFLSEGYAAHLA ALEASHRSRP*
**C1ab-Prkd1**	*X.leavis* map2k1.L^32-44^-mCherry-*M. musculus* Prkd1^138-343^	(Kim et al., 2011)	MALQKKLEELELDEAPVATMVSKGEEDNMAIIKEFMRFKVHMEGS VNGHEFEIEGEGEGRPYEGTQTAKLKVTKGGPLPFAWDILSPQFM YGSKAYVKHPADIPDYLKLSFPEGFKWERVMNFEDGGVVTVTQDS SLQDGEFIYKVKLRGTNFPSDGPVMQKKTMGWEASSERMYPEDG ALKGEIKQRLKLKDGGHYDAEVKTTYKAKKPVQLPGAYNVNIKLDIT SHNEDYTIVEQYERAEGRHSTGGMDELYKSGLRSRAQASNSTSED FQIRPHALFVHSYRAPAFCDHCGEMLWGLVRQGLKCEGCGLNYH KRCAFKIPNNCSGVRRRRLSNVSLTGLGTVRTASAEFSTSVPDEPL LSPVSPGFEQKSPSESFIGREKRSNSQSYIGRPIQLDKLLMSKVKV PHTFVIHSYTRPTVCQFCKKLLKGLFRQGLQCKDCRFNCHKRCAP KVPNNCLGEVTINGELLSPGAES*
**FKBP-PJ-Dead**	mCherry-FKBP1A^3-109^-*S. cerevisiae* Sac1^2-517, C392S^ - INPP5E^214-644, D556A,C641A^	(Hammond et al., 2012)	MVSKGEEDNMAIIKEFMRFKVHMEGSVNGHEFEIEGEGEGRPYEG TQTAKLKVTKGGPLPFAWDILSPQFMYGSKAYVKHPADIPDYLKLS FPEGFKWERVMNFEDGGVVTVTQDSSLQDGEFIYKVKLRGTNFPS DGPVMQKKTMGWEASSERMYPEDGALKGEIKQRLKLKDGGHYDA EVKTTYKAKKPVQLPGAYNVNIKLDITSHNEDYTIVEQYERAEGRHS TGGMDELYKSGLRSRSAAAGAGGAARAALGVQVETISPGDGRTFP KRGQTCVVHYTGMLEDGKKFDSSRDRNKPFKFMLGKQEVIRGWE EGVAQMSVGQRAKLTISPDYAYGATGHPGIIPPHATLVFDVELLKLE SAGGSAGGSAGGSAGGSAGGPRAQASRSLDATGPIVYVQNADGI FFKLAEGKGTNDAVIHLANQDQGVRVLGAEEFPVQGEVVKIASLM GFIKLKLNRYAIIANTVEETGRFNGHVFYRVLQHSIVSTKFNSRIDSE EAEYIKLLELHLKNSTFYFSYTYDLTNSLQRNEKVGPAASWKTADE RFFWNHYLTEDLRNFAHQDPRIDSFIQPVIYGYAKTVDAVLNATPIV LGLITRRSIFRAGTRYFRRGVDKDGNVGNFNETEQILLAENPESEKI HVFSFLQTRGSVPIYWAEINNLKYKPNLVLGENSLDATKKHFDQQK ELYGDNYLVNLVNQKGHELPVKEGYESVVHALNDPKIHYVYFDFH HECRKMQWHRVKLLIDHLEKLGLSNEDFFHKVIDSNGNTVEIVNEQ HSVVRTN[S]MDCLDRTNVVQSVLAQWVLQKEFESADVVATGSTW EDNAPLLTSYQNLWADNADAVSVAYSGTGALKTDFTRTGKRTRLG AFNDFLNSASRYYQNNWTDGPRQDSYDLFLGGFRPHTASIKSPFP GGTARGAAAGAGGAGRSDLADYKLRAQPLLVRAHSSLGPGRPRS PLACDDCSLRSAKSSFSLLAPIRSKDVRSRSYLEGSLLASGALLGA DELARYFPDRNVALFVATWNMQGQKELPPSLDEFLLPAEADYAQD LYVIGVQEGCSDRREWETRLQETLGPHYVLLSSAAHGVLYMSLFIR RDLIWFCSEVECSTVTTRIVSQIKTKGALGISFTFFGTSFLFITSHFTS GDGKVAERLLDYTRTVQALVLPRNVPDTNPYRSSAADVTTRFDEV FWFGDFNFRLSGGRTVVDALLCQGLVVDVPALLQHDQLIREMRKG SIFKGFQEPDIHFLPSYKFDIGKDTYDSTSKQRTPSYT[A]RVLYRSR HKGDICPVSYSSCPGIKTSDHRPVYGLFRVKVRPGRDNIPLAAGKF DRELYLLGIKRRISKEIQRQQALQSQNSSTI[A]SVS*
**FKBP-PJ**	mCherry-FKBP1A^3-109^-*S. cerevisiae* Sac1^2-517^- INPP5E^214-644, C641A^	(Hammond et al., 2012)	MVSKGEEDNMAIIKEFMRFKVHMEGSVNGHEFEIEGEGEGRPYEG TQTAKLKVTKGGPLPFAWDILSPQFMYGSKAYVKHPADIPDYLKLS FPEGFKWERVMNFEDGGVVTVTQDSSLQDGEFIYKVKLRGTNFPS DGPVMQKKTMGWEASSERMYPEDGALKGEIKQRLKLKDGGHYDA EVKTTYKAKKPVQLPGAYNVNIKLDITSHNEDYTIVEQYERAEGRHS TGGMDELYKSGLRSRSAAAGAGGAARAALGVQVETISPGDGRTFP KRGQTCVVHYTGMLEDGKKFDSSRDRNKPFKFMLGKQEVIRGWE EGVAQMSVGQRAKLTISPDYAYGATGHPGIIPPHATLVFDVELLKLE SAGGSAGGSAGGSAGGSAGGPRAQASRSLDATGPIVYVQNADGI FFKLAEGKGTNDAVIHLANQDQGVRVLGAEEFPVQGEVVKIASLM GFIKLKLNRYAIIANTVEETGRFNGHVFYRVLQHSIVSTKFNSRIDSE EAEYIKLLELHLKNSTFYFSYTYDLTNSLQRNEKVGPAASWKTADE RFFWNHYLTEDLRNFAHQDPRIDSFIQPVIYGYAKTVDAVLNATPIV LGLITRRSIFRAGTRYFRRGVDKDGNVGNFNETEQILLAENPESEKI HVFSFLQTRGSVPIYWAEINNLKYKPNLVLGENSLDATKKHFDQQK ELYGDNYLVNLVNQKGHELPVKEGYESVVHALNDPKIHYVYFDFH HECRKMQWHRVKLLIDHLEKLGLSNEDFFHKVIDSNGNTVEIVNEQ HSVVRTNCMDCLDRTNVVQSVLAQWVLQKEFESADVVATGSTWE DNAPLLTSYQNLWADNADAVSVAYSGTGALKTDFTRTGKRTRLGA FNDFLNSASRYYQNNWTDGPRQDSYDLFLGGFRPHTASIKSPFPG GTARGAAAGAGGAGRSDLADYKLRAQPLLVRAHSSLGPGRPRSP LACDDCSLRSAKSSFSLLAPIRSKDVRSRSYLEGSLLASGALLGAD ELARYFPDRNVALFVATWNMQGQKELPPSLDEFLLPAEADYAQDL YVIGVQEGCSDRREWETRLQETLGPHYVLLSSAAHGVLYMSLFIRR DLIWFCSEVECSTVTTRIVSQIKTKGALGISFTFFGTSFLFITSHFTSG DGKVAERLLDYTRTVQALVLPRNVPDTNPYRSSAADVTTRFDEVF WFGDFNFRLSGGRTVVDALLCQGLVVDVPALLQHDQLIREMRKGS IFKGFQEPDIHFLPSYKFDIGKDTYDSTSKQRTPSYTDRVLYRSRHK GDICPVSYSSCPGIKTSDHRPVYGLFRVKVRPGRDNIPLAAGKFDR ELYLLGIKRRISKEIQRQQALQSQNSSTI[A]SVS*
**FKBP-INPP5E**	mCherry-FKBP1A^3-109^-INPP5E^214-644, C641A^	(Hammond et al., 2012)	MVSKGEEDNMAIIKEFMRFKVHMEGSVNGHEFEIEGEGEGRPYEG TQTAKLKVTKGGPLPFAWDILSPQFMYGSKAYVKHPADIPDYLKLS FPEGFKWERVMNFEDGGVVTVTQDSSLQDGEFIYKVKLRGTNFPS DGPVMQKKTMGWEASSERMYPEDGALKGEIKQRLKLKDGGHYDA EVKTTYKAKKPVQLPGAYNVNIKLDITSHNEDYTIVEQYERAEGRHS TGGMDELYKSGLRSRSAAAGAGGAARAAMGVQVETISPGDGRTF PKRGQTCVVHYTGMLEDGKKFDSSRDRNKPFKFMLGKQEVIRGW EEGVAQMSVGQRAKLTISPDYAYGATGHPGIIPPHATLVFDVELLKLE ARGAAAGAGGAGRSDLADYKLRAQPLLVRAHSSLGPGRPRSPL ACDDCSLRSAKSSFSLLAPIRSKDVRSRSYLEGSLLASGALLGADE LARYFPDRNVALFVATWNMQGQKELPPSLDEFLLPAEADYAQDLY VIGVQEGCSDRREWETRLQETLGPHYVLLSSAAHGVLYMSLFIRR DLIWFCSEVECSTVTTRIVSQIKTKGALGISFTFFGTSFLFITSHFTSG DGKVAERLLDYTRTVQALVLPRNVPDTNPYRSSAADVTTRFDEVF WFGDFNFRLSGGRTVVDALLCQGLVVDVPALLQHDQLIREMRKGS IFKGFQEPDIHFLPSYKFDIGKDTYDSTSKQRTPSYTDRVLYRSRHK GDICPVSYSSCPGIKTSDHRPVYGLFRVKVRPGRDNIPLAAGKFDR ELYLLGIKRRISKEIQRQQALQSQNSSTI[A]SVS*
**FKBP-Sac1**	mCherry-FKBP1A^3-109^-*S. cerevisiae* Sac1^2-517^-INPP5E^214-644, D556A C641A^	(Hammond et al., 2012)	MVSKGEEDNMAIIKEFMRFKVHMEGSVNGHEFEIEGEGEGRPYEG TQTAKLKVTKGGPLPFAWDILSPQFMYGSKAYVKHPADIPDYLKLS FPEGFKWERVMNFEDGGVVTVTQDSSLQDGEFIYKVKLRGTNFPS DGPVMQKKTMGWEASSERMYPEDGALKGEIKQRLKLKDGGHYDA EVKTTYKAKKPVQLPGAYNVNIKLDITSHNEDYTIVEQYERAEGRHS TGGMDELYKSGLRSRSAAAGAGGAARAALGVQVETISPGDGRTFP KRGQTCVVHYTGMLEDGKKFDSSRDRNKPFKFMLGKQEVIRGWE EGVAQMSVGQRAKLTISPDYAYGATGHPGIIPPHATLVFDVELLKLE SAGGSAGGSAGGSAGGSAGGPRAQASRSLDATGPIVYVQNADGI FFKLAEGKGTNDAVIHLANQDQGVRVLGAEEFPVQGEVVKIASLM GFIKLKLNRYAIIANTVEETGRFNGHVFYRVLQHSIVSTKFNSRIDSE EAEYIKLLELHLKNSTFYFSYTYDLTNSLQRNEKVGPAASWKTADE RFFWNHYLTEDLRNFAHQDPRIDSFIQPVIYGYAKTVDAVLNATPIV LGLITRRSIFRAGTRYFRRGVDKDGNVGNFNETEQILLAENPESEKI HVFSFLQTRGSVPIYWAEINNLKYKPNLVLGENSLDATKKHFDQQK ELYGDNYLVNLVNQKGHELPVKEGYESVVHALNDPKIHYVYFDFH HECRKMQWHRVKLLIDHLEKLGLSNEDFFHKVIDSNGNTVEIVNEQ HSVVRTNCMDCLDRTNVVQSVLAQWVLQKEFESADVVATGSTWE DNAPLLTSYQNLWADNADAVSVAYSGTGALKTDFTRTGKRTRLGA FNDFLNSASRYYQNNWTDGPRQDSYDLFLGGFRPHTASIKSPFPG GTARGAAAGAGGAGRSDLADYKLRAQPLLVRAHSSLGPGRPRSP LACDDCSLRSAKSSFSLLAPIRSKDVRSRSYLEGSLLASGALLGAD ELARYFPDRNVALFVATWNMQGQKELPPSLDEFLLPAEADYAQDL YVIGVQEGCSDRREWETRLQETLGPHYVLLSSAAHGVLYMSLFIRR DLIWFCSEVECSTVTTRIVSQIKTKGALGISFTFFGTSFLFITSHFTSG DGKVAERLLDYTRTVQALVLPRNVPDTNPYRSSAADVTTRFDEVF WFGDFNFRLSGGRTVVDALLCQGLVVDVPALLQHDQLIREMRKGS IFKGFQEPDIHFLPSYKFDIGKDTYDSTSKQRTPSYT[A]RVLYRSRH KGDICPVSYSSCPGIKTSDHRPVYGLFRVKVRPGRDNIPLAAGKFD RELYLLGIKRRISKEIQRQQALQSQNSSTI[A]SVS*
**PM-FRB**	Lyn^1-11^-MTOR^2021-2113^-iRFP	(Hammond et al., 2014)	MGCIKSKGKDSRSANSGAGAGAGAILSRILWHEMWHEGLEEASRL YFGERNVKGMFEVLEPLHAMMERGPQTLKETSFNQAYGRDLMEA QEWCRKYMKSGNVKDLTQAWDLYYHVFRRISKTSYPYDVPDYAP VATMAEGSVARQPDLLTCDDEPIHIPGAIQPHGLLLALAADMTIVAG SDNLPELTGLAIGALIGRSAADVFDSETHNRLTIALAEPGAAVGAPIT VGFTMRKDAGFIGSWHRHDQLIFLELEPPQRDVAEPQAFFRRTNS AIRRLQAAETLESACAAAAQEVRKITGFDRVMIYRFASDFSGEVIAE DRCAEVESKLGLHYPASTVPAQARRLYTINPVRIIPDINYRPVPVTP DLNPVTGRPIDLSFAILRSVSPVHLEFMRNIGMHGTMSISILRGERL WGLIVCHHRTPYYVDLDGRQACELVAQVLAWQIGVMEE*
**Mito-FRB**	iRFP-MTOR^2021-2113^-Fis^122-152^	(Doyle et al., 2023)	MAEGSVARQPDLLTCDDEPIHIPGAIQPHGLLLALAADMTIVAGSDN LPELTGLAIGALIGRSAADVFDSETHNRLTIALAEPGAAVGAPITVGF TMRKDAGFIGSWHRHDQLIFLELEPPQRDVAEPQAFFRRTNSAIRR LQAAETLESACAAAAQEVRKITGFDRVMIYRFASDFSGEVIAEDRC AEVESKLGLHYPASTVPAQARRLYTINPVRIIPDINYRPVPVTPDLNP VTGRPIDLSFAILRSVSPVHLEFMRNIGMHGTMSISILRGERLWGLIV CHHRTPYYVDLDGRQACELVAQVLAWQIGVMEESGLRSRAGGAG AILSRILWHEMWHEGLEEASRLYFGERNVKGMFEVLEPLHAMMER GPQTLKETSFNQAYGRDLMEAQEWCRKYMKSGNVKDLTQAWDL YYHVFRRISKGGSAGGSAQASNSAVDGTAGLVGMAIVGGMALGV AGLAGLIGLAVSKSKS*
**FKBP-PI4K**	mCherry-FKBP1A^3-108^-PI4KA^1102-2103^	(Zewe et al., 2020)	MVSKGEEDNMAIIKEFMRFKVHMEGSVNGHEFEIEGEGEGRPYEG TQTAKLKVTKGGPLPFAWDILSPQFMYGSKAYVKHPADIPDYLKLS FPEGFKWERVMNFEDGGVVTVTQDSSLQDGEFIYKVKLRGTNFPS DGPVMQKKTMGWEASSERMYPEDGALKGEIKQRLKLKDGGHYDA EVKTTYKAKKPVQLPGAYNVNIKLDITSHNEDYTIVEQYERAEGRHS TGGMDELYKSGLRSRSAAAGAGGAARAALGVQVETISPGDGRTFP KRGQTCVVHYTGMLEDGKKFDSSRDRNKPFKFMLGKQEVIRGWE EGVAQMSVGQRAKLTISPDYAYGATGHPGIIPPHATLVFDVELLKLE SAGGSAGGSAGGSAGGSAGGPRAQASNSMATESILHFAGYNKQN TTLGATQLSERPACVKKDYSNFMASLNLRNRYAGEVYGMIRFSGT TGQMSDLNKMMVQDLHSALDRSHPQHYTQAMFKLTAMLISSKDC DPQLLHHLCWGPLRMFNEHGMETALACWEWLLAGKDGVEVPFM REMAGAWHMTVEQKFGLFSAEIKEADPLAASEASQPKPCPPEVTP HYIWIDFLVQRFEIAKYCSSDQVEIFSSLLQRSMSLNIGGAKGSMNR HVAAIGPRFKLLTLGLSLLHADVVPNATIRNVLREKIYSTAFDYFSCP PKFPTQGEKRLREDISIMIKFWTAMFSDKKYLTASQLVPPDNQDTR SNLDITVGSRQQATQGWINTYPLSSGMSTISKKSGMSKKTNRGSQ LHKYYMKRRTLLLSLLATEIERLITWYNPLSAPELELDQAGENSVAN WRSKYISLSEKQWKDNVNLAWSISPYLAVQLPARFKNTEAIGNEVT RLVRLDPGAVSDVPEAIKFLVTWHTIDADAPELSHVLCWAPTDPPT GLSYFSSMYPPHPLTAQYGVKVLRSFPPDAILFYIPQIVQALRYDKM GYVREYILWAASKSQLLAHQFIWNMKTNIYLDEEGHQKDPDIGDLL DQLVEEITGSLSGPAKDFYQREFDFFNKITNVSAIIKPYPKGDERKK ACLSALSEVKVQPGCYLPSNPEAIVLDIDYKSGTPMQSAAKAPYLA KFKVKRCGVSELEKEGLRCRSDSEDECSTQEADGQKISWQAAIFK VGDDCRQDMLALQIIDLFKNIFQLVGLDLFVFPYRVVATAPGCGVIE CIPDCTSRDQLGRQTDFGMYDYFTRQYGDESTLAFQQARYNFIRS MAAYSLLLFLLQIKDRHNGNIMLDKKGHIIHIDFGFMFESSPGDNLG WEPDIKLTDEMVMIMGGKMEATPFKWFMEMCVRGYLAVRPYMDA VVSLVTLMLDTGLPCFRGQTIKLLKHRFSPNMTEREAANFIMKVIQS CFLSNRSRTYDMIQYYQNDIPY*
**FKBP-PIP5K**	pTagBFP-FKBP1A^3-108^-PIP5K1C^1-635, D101R, R304D, R445E, K446E^	This Study	MVSELIKENMHMKLYMEGTVDNHHFKCTSEGEGKPYEGTQTMRIK VVEGGPLPFAFDILATSFLYGSKTFINHTQGIPDFFKQSFPEGFTWE RVTTYEDGGVLTATQDTSLQDGCLIYNVKIRGVNFTSNGPVMQKKT LGWEAFTETLYPADGGLEGRNDMALKLVGGSHLIANAKTTYRSKK PAKNLKMPGVYYVDYRLERIKEANNETYVEQHEVAVARYCDLPSK LGHKLNSGLRSRSAAAGAGGAARAALGVQVETISPGDGRTFPKRG QTCVVHYTGMLEDGKKFDSSRDRNKPFKFMLGKQEVIRGWEEGV AQMSVGQRAKLTISPDYAYGATGHPGIIPPHATLVFDVELLKLESAG GSAGGSAGGSAGGSAGGPRAQASNSAVDLQAMELEVPDEAESA EAGAVPSEAAWAAESGAAAGLAQKKAAPTEVLSMTAQPGPGHGK KLGHRGVDASGETTYKKTTSSTLKGAIQLGIGYTVGHLSSKPER[R] VLMQDFYVVESIFFPSEGSNLTPAHHFQDFRFKTYAPVAFRYFREL FGIRPDDYLYSLCNEPLIELSNPGASGSLFYVTSDDEFIIKTVMHKEA EFLQKLLPGYYMNLNQNPRTLLPKFYGLYCVQSGGKNIRVVVMNNI LPRVVKMHLKFDLKGSTYKRRASKKEKEKSFPTYKDLDFMQDMPE GLLLDADTFSALVKTLQ[D]DCLVLESFKIMDYSLLLGVHNIDQHERE RQAQGAQSTSDEKRPVGQKALYSTAMESIQGGAARGEAIESDDT MGGIPAVNGRGERLLLHIGIIDILQSYRFIKKLEHTWKALVHDGDTVS VHRPSFYAERFFKFMSNTVF[EE]NSSLKSSPSKKGRGGALLAVKPL GPTAAFSASQIPSEREEAQYDLRGARSYPTLEDEGRPDLLPCTPPS FEEATTASIATTLSSTSLSIPERSPSETSEQPRYRRRTQSSGQDGR PQEEPPAEEDLQQITVQVEPACSVEIVVPKEEDAGVEASPAGASAA VEVETASQASDEEGAPASQASDEEDAPATDIYF*
**EGFP-PH-PLCd1**	PLCd1^1-170^-EGFP	(Várnai and Balla, 1998)	MDSGRDFLTLHGLQDDEDLQALLKGSQLLKVKSSSWRRERFYKLQ EDCKTIWQESRKVMRTPESQLFSIEDIQEVRMGHRTEGLEKFARD VPEDRCFSIVFKDQRNTLDLIAPSPADAQHWVLGLHKIIHHSGSMD QRQKLQHWIHSCLRKADKNKDNKMSFKELQNFLKDPPVATMVSK GEELFTGVVPILVELDGDVNGHKFSVSGEGEGDATYGKLTLKFICT TGKLPVPWPTLVTTLTYGVQCFSRYPDHMKQHDFFKSAMPEGYV QERTIFFKDDGNYKTRAEVKFEGDTLVNRIELKGIDFKEDGNILGHK LEYNYNSHNVYIMADKQKNGIKVNFKIRHNIEDGSVQLADHYQQNT PIGDGPVLLPDNHYLSTQSALSKDPNEKRDHMVLLEFVTAAGITLG MDELYK*
**FKBP-PI-PLC**	*B. cereus* PI-PLC^32-329, W78A, W273A^-FKBP1A^3-108^-TagBFP2	(Pemberton et al., 2020)	MASSVNELENWSKWMQPIPDNIPLARISIPGTHDSGTFKLQNPIKQ V[A]GMTQEYDFRYQMDHGARIFDIRGRLTDDNTIVLHHGPLYLYVT LHEFINEAKQFLRDNPSETIIMSLKKEYEDMKGAEDSFSSTFEKNYF VDPIFLKTEGNIKLGDARGKIVLLKRYSGSNESGGYNNFYWPDNET FTTTVNQNVNVTVQDKYKVSYDEKVKSIKDTMNETMNNSEDLNHL YINFTSLSSGGTA[A]NSPYYYASYINPEIANHIKQKNPARVGWVIQD YINEKWSPLLYQEVIRANKSLIKERILQSTVPMGVQVETISPGDGRT FPKRGQTCVVHYTGMLEDGKKFDSSRDRNKPFKFMLGKQEVIRG WEEGVAQMSVGQRAKLTISPDYAYGATGHPGIIPPHATLVFDVELL KLERDPPVATMVSELIKENMHMKLYMEGTVDNHHFKCTSEGEGKP YEGTQTMRIKVVEGGPLPFAFDILATSFLYGSKTFINHTQGIPDFFK QSFPEGFTWERVTTYEDGGVLTATQDTSLQDGCLIYNVKIRGVNFT SNGPVMQKKTLGWEAFTETLYPADGGLEGRNDMALKLVGGSHLIA NAKTTYRSKKPAKNLKMPGVYYVDYRLERIKEANNETYVEQHEVA VARYCDLPSKLGHKLNSGLRSRSAAATLDHNQPYHICRGFTCFKK PPTPPPEPET*
**FKBP-DGKα**	mRFP-FKBP1A^3-108^-DGKA isoform b^394-773^	This Study	MASSEDVIKEFMRFKVRMEGSVNGHEFEIEGEGEGRPYEGTQTAK LKVTKGGPLPFAWDILSPQFQYGSKAYVKHPADIPDYLKLSFPEGF KWERVMNFEDGGVVTVTQDSSLQDGEFIYKVKLRGTNFPSDGPV MQKKTMGWEASTERMYPEDGALKGEIKMRLKLKDGGHYDAEVKT TYMAKKPVQLPGAYKTDIKLDITSHNEDYTIVEQYERAEGRHSTGA SGLRSRSAAAGAGGAARAALGVQVETISPGDGRTFPKRGQTCVV HYTGMLEDGKKFDSSRDRNKPFKFMLGKQEVIRGWEEGVAQMSV GQRAKLTISPDYAYGATGHPGIIPPHATLVFDVELLKLESAGGSAGG SAGGSAGGSAGGPRAQASRSDDLNLSTSEALRIDPVPNTHPLLVF VNPKSGGKQGQRVLWKFQYILNPRQVFNLLKDGPEIGLRLFKDVP DSRILVCGGDGTVGWILETIDKANLPVLPPVAVLPLGTGNDLARCLR WGGGYEGQNLAKILKDLEMSKVVHMDRWSVEVIPQQTEEKSDPV PFQIINNYFSIGVDASIAHRFHIMREKYPEKFNSRMKNKLWYFEFAT SESIFSTCKKLEESLTVEICGKPLDLSNLSLEGIAVLNIPSMHGGSNL WGDTRRPHGDIYGINQALGATAKVITDPDILKTCVPDLSDKRLEVV GLEGAIEMGQIYTKLKNAGRRLAKCSEITFHTTKTLPMQIDGEPWM QTPCTIKITHKNQMPMLMGPPPRSTNFFGFLS*
**M3**	pcDNA3.1-HAx3-AchR-CHRM3^2-590^	J. Wess	MYPYDVPDYAYPYDVPDYAYPYDVPDYADTLHNNSTTSPLFPNISS SWIHSPSDAGLPPGTVTHFGSYNVSRAAGNFSSPDGTTDDPLGGH TVWQVVFIAFLTGILALVTIIGNILVIVSFKVNKQLKTVNNYFLLSLACA DLIIGVISMNLFTTYIIMNRWALGNLACDLWLAIDYVASNASVMNLLV ISFDRYFSITRPLTYRAKRTTKRAGVMIGLAWVISFVLWAPAILFWQ YFVGKRTVPPGECFIQFLSEPTITFGTAIAAFYMPVTIMTILYWRIYK ETEKRTKELAGLQASGTEAETENFVHPTGSSRSCSSYELQQQSMK RSNRRKYGRCHFWFTTKSWKPSSEQMDQDHSSSDSWNNNDAAA SLENSASSDEEDIGSETRAIYSIVLKLPGHSTILNSTKLPSSDNLQVP EEELGMVDLERKADKLQAQKSVDDGGSFPKSFSKLPIQLESAVDT AKTSDVNSSVGKSTATLPLSFKEATLAKRFALKTRSQITKRKRMSLV KEKKAAQTLSAILLAFIITWTPYNIMVLVNTFCDSCIPKTFWNLGYWL CYINSTVNPVCYALCNKTFRTTFKMLLLCQCDKKKRRKQQYQQRQ SVIFHKRAPEQAL*
**GFP-Nir2**	EGFP-PITPNM1v2	(Kim et al., 2015)	MVSKGEELFTGVVPILVELDGDVNGHKFSVSGEGEGDATYGKLTL KFICTTGKLPVPWPTLVTTLTYGVQCFSRYPDHMKQHDFFKSAMP EGYVQERTIFFKDDGNYKTRAEVKFEGDTLVNRIELKGIDFKEDGNI LGHKLEYNYNSHNVYIMADKQKNGIKVNFKIRHNIEDGSVQLADHY QQNTPIGDGPVLLPDNHYLSTQSALSKDPNEKRDHMVLLEFVTAA GITLGMDELYKSGLRSRAQASNSMLIKEYHILLPMSLDEYQVAQLY MIQKKSREESSGEGSGVEILANRPYTDGPGGSGQYTHKVYHVGSH IPGWFRALLPKAALQVEEESWNAYPYTRTRYTCPFVEKFSIEIETYY LPDGGQQPNVFNLSGAERRQRILDTIDIVRDAVAPGEYKAEEDPRL YHSVKTGRGPLSDDWARTAAQTGPLMCAYKLCKVEFRYWGMQA KIEQFIHDVGLRRVMLRAHRQAWCWQDEWTELSMADIRALEEETA RMLAQRMAKCNTGSEGSEAQPPGKPSTEARSAASNTGTPDGPEA PPGPDASPDASFGKQWSSSSRSSYSSQHGGAVSPQSLSEWRMQ NIARDSENSSEEEFFDAHEGFSDSEEVFPKEMTKWNSNDFIDAFAS PVEAEGTPEPGAEAAKGIEDGAQAPRDSEGLDGAGELGAEACAVH ALFLILHSGNILDSGPGDANSKQADVQTLSSAFEAVTRIHFPEALGH VALRLVPCPPICAAAYALVSNLSPYSHDGDSLSRSQDHIPLAALPLL ATSSSRYQGAVATVIARTNQAYSAFLRSPEGAGFCGQVALIGDGV GGILGFDALCHSANAGTGSRGSSRRGSMNNELLSPEFGPVRDPLA DGVEGLGRGSPEPSALPPQRIPSDMASPEPEGSQNSLQAAPATTS SWEPRRASTAFCPPAASSEAPDGPSSTARLDFKVSGFFLFGSPLG LVLALRKTVMPALEAQMRPACEQIYNLFHAADPCASRLEPLLAPKF QAIAPLTVPRYQKFPLGDGSSLLLADTLQTHSSLFLEELEMLVPSTP TSTSGAFWKGSELATDPPAQPAAPSTTSEVVKILERWWGTKRIDY SLYCPEALTAFPTVTLPHLFHASYWESADVVAFILRQVIEKERPQLA ECEEPSIYSPAFPREKWQRKRTQVKIRNVTSNHRASDTVVCEGRP QVLSGRFMYGPLDVVTLTGEKVDVYIMTQPLSGKWIHFGTEVTNS SGRLTFPVPPERALGIGVYPVRMVVRGDHTYAECCLTVVARGTEA VVFSIDGSFTASVSIMGSDPKVRAGAVDVVRHWQDSGYLIVYVTG RPDMQKHRVVAWLSQHNFPHGVVSFCDGLTHDPLRQKAMFLQSL VQEVELNIVAGYGSPKDVAVYAALGLSPSQTYIVGRAVRKLQAQCQ FLSDGYVAHLGQLEAGSHSHASSGPPRAALGKSSYGVAAPVDFLR KQSQLLRSRGPSQAEREGPGTPPTTLARGKARSISLKLDSEE*
**iRFP-Nir1-LNS2**	miRFP670-Nir1^613-897^	This Study	MVAGHASGSPAFGTASHSNCEHEEIHLAGSIQPHGALLVVSEHDH RVIQASANAAEFLNLGSVLGVPLAEIDGDLLIKILPHLDPTAEGMPVA VRCRIGNPSTEYCGLMHRPPEGGLIIELERAGPSIDLSGTLAPALERI RTAGSLRALCDDTVLLFQQCTGYDRVMVYRFDEQGHGLVFSECH VPGLESYFGNRYPSSTVPQMARQLYVRQRVRVLVDVTYQPVPLEP RLSPLTGRDLDMSGCFLRSMSPCHLQFLKDMGVRATLAVSLVVGG KLWGLVVCHHYLPRFIRFELRAICKRLAERIATRITALESGGSGGMP ANPREKWLRKRTQVKLRNVTANHRANDVIAAEDGPQVLVGRFMY GPLDMVALTGEKVDILVMAEPSSGRWVHLDTEITNSSGRITYNVPR PRRLGVGVYPVKMVVRGDQTCAMSYLTVLPRGMECVVFSIDGSFA ASVSIMGSDPKVRPGAVDVVRHWQDLGYMILYITGRPDMQKQRVV SWLSQHNFPQGMIFFSDGLVHDPLRQKAIFLRNLMQECFIKISAAY GSTKDISVYSVLGLPASQIFIVGRPTKKYQTQCQFLSEGYAAHLAAL EASHRSRPKKN*
**iRFP-Tubby**	*M. musculus* Tubby^243-505^-miRFP670	(Quinn et al., 2008)	MVDIEVQDLEEFALRPAPQGITIKCRITRDKKGMDRGMYPTYFLHL DREDGKKVFLLAGRKRKKSKTSNYLISVDPTDLSRGGDSYIGKLRS NLMGTKFTVYDNGVNPQKASSSTLESGTLRQELAAVCYETNVLGF KGPRKMSVIVPGMNMVHERVCIRPRNEHETLLARWQNKNTESIIEL QNKTPVWNDDTQSYVLNFHGRVTQASVKNFQIIHGNDPDYIVMQF GRVAEDVFTMDYNYPLCALQAFAIALSSFDSKLACETVPRARDPPV ATMVAGHASGSPAFGTASHSNCEHEEIHLAGSIQPHGALLVVSEH DHRVIQASANAAEFLNLGSVLGVPLAEIDGDLLIKILPHLDPTAEGMP VAVRCRIGNPSTEYCGLMHRPPEGGLIIELERAGPSIDLSGTLAPAL ERIRTAGSLRALCDDTVLLFQQCTGYDRVMVYRFDEQGHGLVFSE CHVPGLESYFGNRYPSSTVPQMARQLYVRQRVRVLVDVTYQPVP LEPRLSPLTGRDLDMSGCFLRSMSPCHLQFLKDMGVRATLAVSLV VGGKLWGLVVCHHYLPRFIRFELRAICKRLAERIATRITALES*
**iRFP-PH-PLCδ1**	iRFP713-*R. norvegicus* PLCD1^2-131^	(Idevall-Hagren et al., 2012)	MAEGSVARQPDLLTCDDEPIHIPGAIQPHGLLLALAADMTIVAGSDN LPELTGLAIGALIGRSAADVFDSETHNRLTIALAEPGAAVGAPITVGF TMRKDAGFIGSWHRHDQLIFLELEPPQRDVAEPQAFFRRTNSAIRR LQAAETLESACAAAAQEVRKITGFDRVMIYRFASDFSGEVIAEDRC AEVESKLGLHYPASTVPAQARRLYTINPVRIIPDINYRPVPVTPDLNP VTGRPIDLSFAILRSVSPVHLEFMRNIGMHGTMSISILRGERLWGLIV CHHRTPYYVDLDGRQACELVAQVLAWQIGVMEECTRDLELKLHGL QDDPDLQALLKGSQLLKVKSSSWRRERFYKLQEDCKTIWQESRKV MRSPESQLFSIEDIQEVRMGHRTEGLEKFARDIPEDRCFSIVFKDQ RNTLDLIAPSPADAQHWVQGLRKIIHHSGSMDQRQK*
**TagBFP2-CAAX**	TagBFP2-HRAS^172-189^	(Goulden et al., 2019)	MVSELIKENMHMKLYMEGTVDNHHFKCTSEGEGKPYEGTQTMRIK VVEGGPLPFAFDILATSFLYGSKTFINHTQGIPDFFKQSFPEGFTWE RVTTYEDGGVLTATQDTSLQDGCLIYNVKIRGVNFTSNGPVMQKKT LGWEAFTETLYPADGGLEGRNDMALKLVGGSHLIANAKTTYRSKK PAKNLKMPGVYYVDYRLERIKEANNETYVEQHEVAVARYCDLPSK LGHKLNSGLRSRAQASNSAVDNPPDESGPGCMSCKCVLS*
