# Supplementary material for: PILS-Nir1 is a novel phosphatidic acid biosensor that reveals mechanisms of lipid production
Source: bioRxiv. 2025 Jul 10:2024.02.28.582557. Originally published 2024 Feb 28. Preprint. [Version 2] doi: 10.1101/2024.02.28.582557 (PMC10925316; doi:10.1101/2024.02.28.582557)
Supplement: 1 [file NIHPP2024.02.28.582557V2-supplement-1.pdf]

bioRxiv preprint doi: https://doi.org/10.1101/2024.02.28.582557; this version posted July 10, 2025. The copyright holder for this preprint (which was not certified by peer review) is the author/funder, who has granted bioRxiv a license to display the preprint in perpetuity. It is made available under aCC-BY 4.0 International license.

	NES-PABD	PASS	NES-flex-PABD	NES-PABDx2	NESx2-PABDx2	PILS-Nir1	Nir2-LNS2
PASS<br>μ = 1.830	0.9999						
NES-flex-PABD<br>μ = 1.052	>0.9999	0.9999					
NES-PABDx2<br>μ = 4.195	0.7860	0.9435	0.7475				
NESx2-PABDx2<br>μ = 4.429	0.7251	0.9114	0.6791	>0.9999			
PILS-Nir1<br>μ = 15.42	<0.0001	<0.0001	<0.0001	0.0004	0.0005		
Nir2-LNS2<br>μ = 6.334	0.1774	0.3493	0.1289	0.9523	0.9739	0.0017	
Nir3-LNS2<br>μ = 3.329	0.9460	0.9955	0.9350	0.9999	0.9994	0.0002	0.7853

Table S1. P-values from the ordinary one-way ANOVA with multiple comparisons for AUC biosensor data presented in **Figure 1J**. The mean AUC value for each biosensor is shown on the left as "μ". Significant p-values ($p < 0.05$) are highlighted in gray.

bioRxiv preprint doi: https://doi.org/10.1101/2024.02.28.582557; this version posted July 10, 2025. The copyright holder for this preprint (which was not certified by peer review) is the author/funder, who has granted bioRxiv a license to display the preprint in perpetuity. It is made available under aCC-BY 4.0 International license.

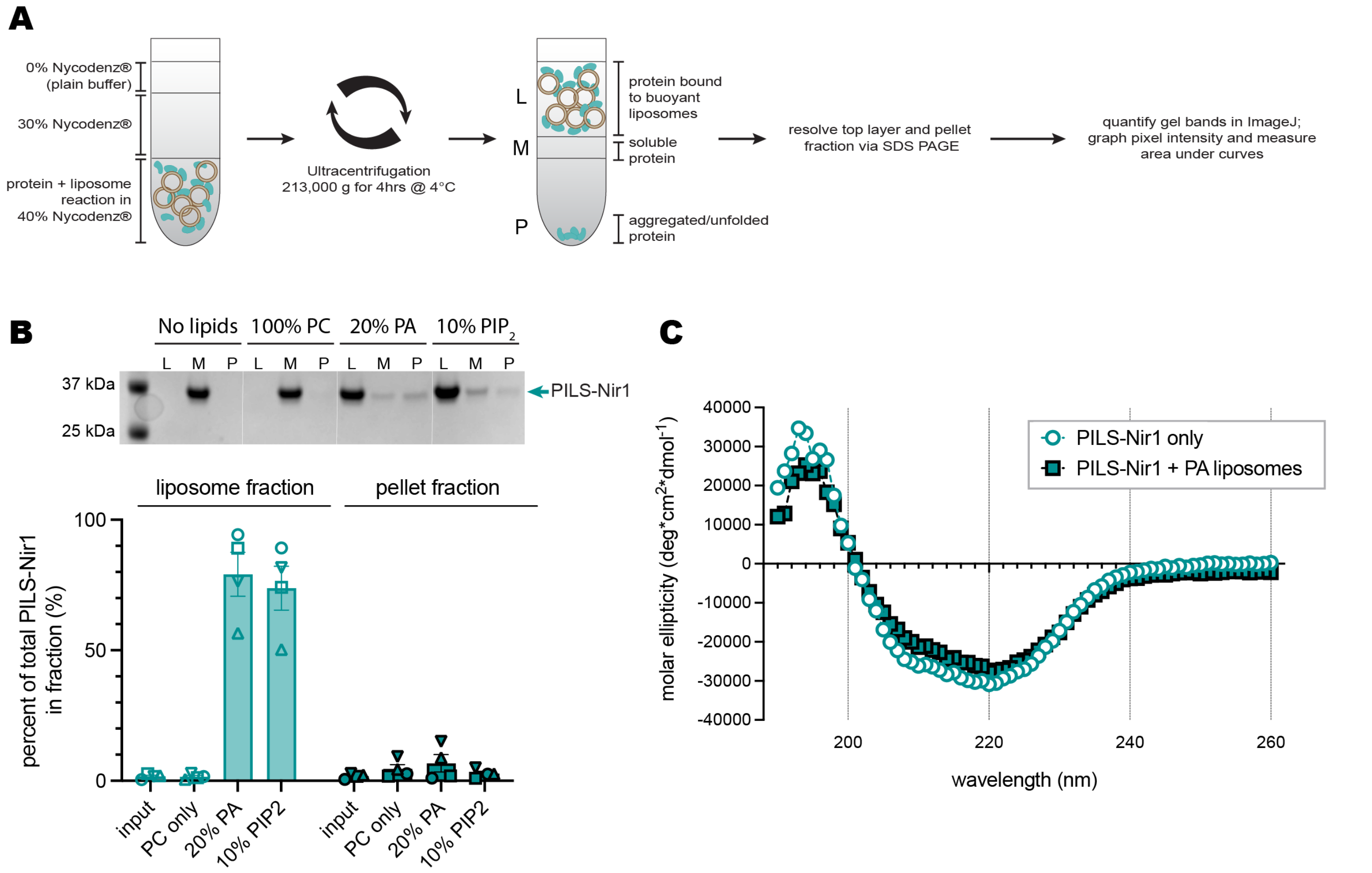

Supplemental Figure 1. PILS-Nir1 does not aggregate or unfold in *in vitro* liposome experiments. (A) Schematic of PILS-Nir1 liposome flotation assay. **(B)** Representative SDS-PAGE gel and quantification of PILS-Nir1 bound to liposomes in the liposome fraction (L), as soluble protein in the middle fraction (M), or as aggregated protein in the pellet fraction (P) after reacting with POPC liposomes of varying compositions. **(C)** Circular dichroism for PILS-Nir1 with and without PA liposomes.

bioRxiv preprint doi: https://doi.org/10.1101/2024.02.28.582557; this version posted July 10, 2025. The copyright holder for this preprint (which was not certified by peer review) is the author/funder, who has granted bioRxiv a license to display the preprint in perpetuity. It is made available under aCC-BY 4.0 International license.

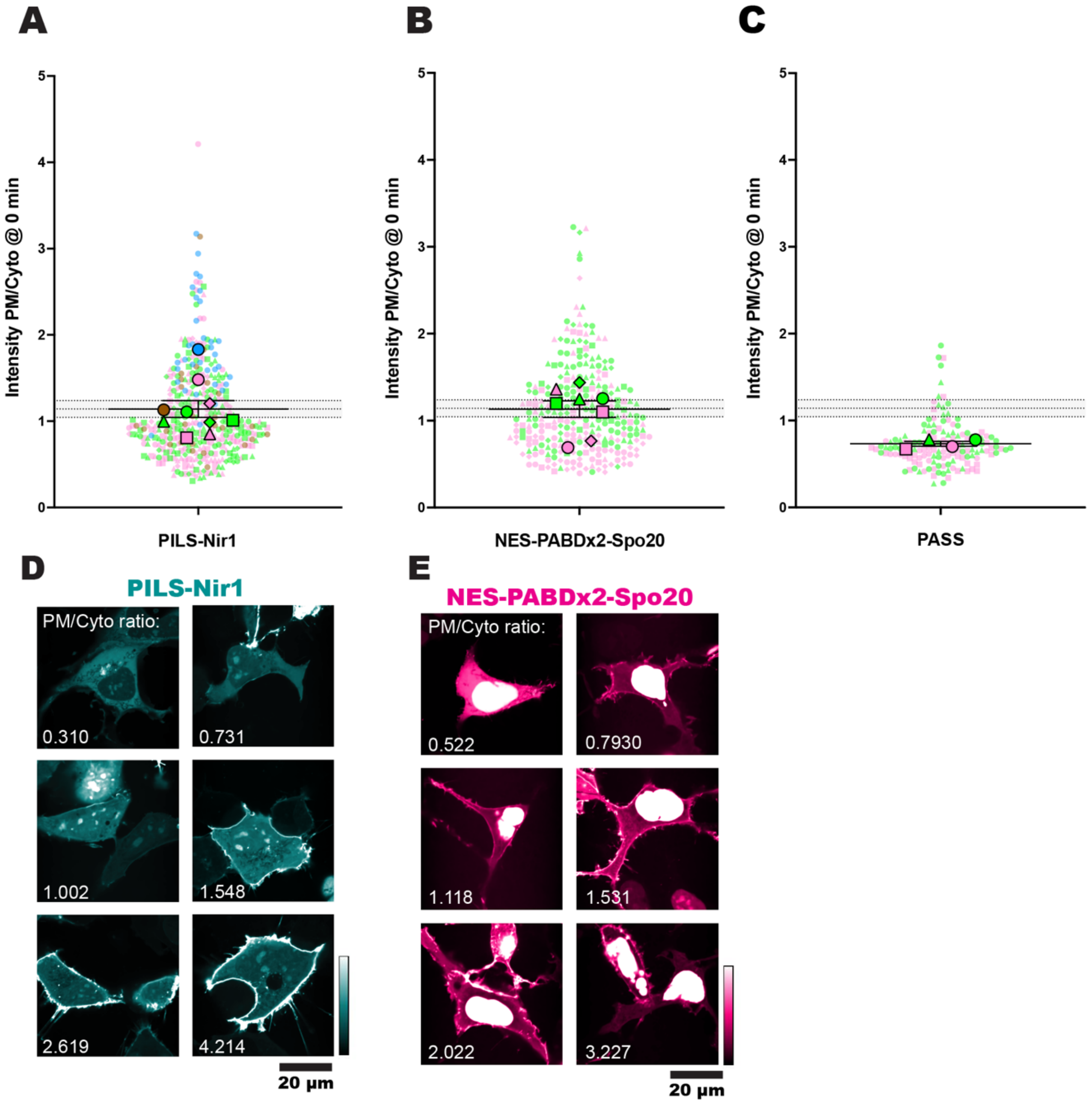

Supplemental Figure 2. PA biosensors can associate with the PM under resting conditions. Basal localization of PILS-Nir1 **(A)**, NES-PABDx2-Spo20 **(B)**, and PASS **(C)**. Each small symbol represents the biosensor intensity PM/Cyto ratio of a single cell at time 0 min, before any treatment was added (n = 418 PILS-Nir1 cells, 288 NES-PABDx2-Spo20 cells, and 74 PASS cells). The large symbols show the grand means of each experimental replicate (n = 3-6 independent experiments). The symbols are color coded according to the figure where the data can be found. Pink cells are in Figure 1. Blue cells are in Figure 2. Green cells are in Figure 7. Brown cells are cells co-expressing FKBP-PJ-Dead (a catalytically dead PIP phosphatase used as a control in Figure 3). The shape of the symbol denotes different dishes within each experiment (i.e cells that were to be treated with PMA or cells that were to be treated with PMA + FIPI). Note that not all treatments shown here were included in their respective figures. Error bars show the mean ± SEM. The gray shaded area shows the PILS-Nir1 grand mean ± SEM to facilitate comparison between graphs. Representative confocal images of PILS-Nir1 **(D)** and NES-PABDx2-Spo20 **(E)** show the range of basal PM/Cyto ratios seen across these experiments, with the given ratio values labelled on each image.
