# Supplementary material for: Nir1-LNS2 is a novel phosphatidic acid biosensor that reveals mechanisms of lipid production
Source: bioRxiv. 2024 Feb 28:2024.02.28.582557. Preprint. [Version 1] doi: 10.1101/2024.02.28.582557 (PMC10925316; doi:10.1101/2024.02.28.582557)
Supplement: Supplementary file 1 [file NIHPP2024.02.28.582557v1-supplement-1.pdf]

bioRxiv preprint doi: https://doi.org/10.1101/2024.02.28.582557; this version posted February 28, 2024. The copyright holder for this preprint (which was not certified by peer review) is the author/funder, who has granted bioRxiv a license to display the preprint in perpetuity. It is made available under aCC-BY 4.0 International license.

	NES-PABD	PASS	NES-flex-PABD	NES-PABDx2	NESx2-PABDx2	Nir1-LNS2	Nir2-LNS2
PASS<br>μ = 1.830	0.9999						
NES-flex-PABD<br>μ = 1.052	>0.9999	0.9999					
NES-PABDx2<br>μ = 4.195	0.7860	0.9435	0.7475				
NESx2-PABDx2<br>μ = 4.429	0.7251	0.9114	0.6791	>0.9999			
Nir1-LNS2<br>μ = 15.42	<0.0001	<0.0001	<0.0001	0.0004	0.0005		
Nir2-LNS2<br>μ = 6.334	0.1774	0.3493	0.1289	0.9523	0.9739	0.0017	
Nir3-LNS2<br>μ = 3.329	0.9460	0.9955	0.9350	0.9999	0.9994	0.0002	0.7853

Table S1. P values from the ordinary one-way ANOVA with multiple comparisons for AUC biosensor data presented in **Figure 1J**. The mean AUC value for each biosensor is shown on the left as "μ". Significant p-values ($p < 0.05$) are highlighted in gray.
